# Evaluating the Cost-Effectiveness of Air Pollution Mitigation Strategies: A Systematic Review

**DOI:** 10.3390/ijerph22060926

**Published:** 2025-06-11

**Authors:** Bo Vandenbulcke, Nick Verhaeghe, Lisa Cruycke, Max Lelie, Steven Simoens, Koen Putman

**Affiliations:** 1 Department of Public Health, Interuniversity Centre for Health Economics Research (i-CHER), Vrije Universiteit Brussel, Laarbeeklaan 103, 1090 Jette, Belgium; nick.verhaeghe@vub.be (N.V.); lisa.cruycke@vub.be (L.C.); max.lelie@vub.be (M.L.); koen.putman@vub.be (K.P.); 2 Department of Pharmaceutical and Pharmacological Sciences, KU Leuven, ON2 Herestraat 49, P.O. Box 521, 3000 Leuven, Belgium; steven.simoens@kuleuven.be; 3 Department of Public Health and Primary Care, Interuniversity Centre for Health Economics Research (i-CHER), Ghent University, Corneel Heymanslaan 10, 9000 Ghent, Belgium

**Keywords:** health economics, air pollution, cost-effectiveness, cost–benefit, cost–utility, systematic review

## Abstract

Air pollution is the world’s greatest environmental health risk. Pollutants that pose large health concerns are particulate matter (PM_2.5_ and PM_10_), ozone (O_3_), carbon monoxide (CO), nitrogen dioxide (NO_2_), and sulphur dioxide (SO_2_). These compounds (especially PM_2.5_ and PM_10_) contribute to the onset of diseases, such as respiratory diseases (e.g., asthma, chronic obstructive pulmonary disease (COPD), or lung cancer) and cardiovascular diseases. Abatement interventions are implemented to reduce air pollution and thereby the risk of these diseases. This systematic review examined the published scientific evidence on the cost-effectiveness of strategies aimed at reducing or controlling air pollution and assessed the reporting quality of included studies. It employed rigorous data extraction and quality scoring procedures to ensure the reliability and validity of our findings. Overall, there is substantial evidence supporting the cost-effectiveness of interventions aimed at reducing air pollution. Seventy-four studies and four policy reports were included in this review. Predominantly, cost–benefit analyses have been reported (*n* = 40), reflecting the multisectoral impacts and associated costs of air pollution. Only four cost–utility analyses were found, indicating the need for more research within this domain. Additionally, eight articles reported one or more non-economic results. Thirty-nine studies focused on particulate matter (PM), and eleven focused on nitrogen oxides (NOx). The quality assessment yielded moderate results. The heterogeneity of studies and moderate quality of reporting make it difficult to compare results and draw definitive conclusions.

## 1. Introduction

Clean air is an essential element in making life on Earth possible. However, anthropogenic sources are releasing large amounts of polluting waste into the atmosphere, resulting in air pollution [1]. Air pollution can be defined as “contamination of the indoor or outdoor environment by any chemical, physical or biological agent that modifies the natural characteristics of the atmosphere”. It is the world’s greatest environmental health risk [2,3]. Especially low- and middle-income countries are suffering from this. In large cities, however, exposure to pollutants can also be very high [2,3,4].

The World Health Organization (WHO) differentiates air pollution in ambient air pollution and household air pollution. The first, which includes ozone, is mainly caused by vehicles and industry emissions. The second is generated by cooking with solid fuels, heating homes with wood or coal, and smoking [5,6].

In 2019, air pollution caused 6.7 million deaths worldwide [7]. In Europe, it has been estimated to account for more than 400,000 premature deaths per year [2]. Pollutants that pose large health concerns are particulate matter (PM_2.5_ and PM_10_), ozone, carbon monoxide (CO), nitrogen dioxide (NO_2_), and sulphur dioxide (SO_2_) [3]. These compounds (especially PM_2.5_ and PM_10_) contribute to the onset of diseases, such as respiratory diseases (e.g., asthma, chronic obstructive pulmonary disease (COPD), or lung cancer) and cardiovascular diseases [8]. Long-term exposure to PM_2.5_ is accountable for the loss of 103.1 million disability-adjusted life years (DALYs), which is 7.6% of the overall global DALYs [9].

The economic impact of air pollution is significant. In Brazil, Miraglia et al. (2014) calculated that air pollution costs USD 1.7 billion annually due to premature mortality in urban areas [10]. Shamsi et al. (2021) estimated the health costs resulting from traffic on Highway 401 in Canada at 416 million Canadian dollars per year [11]. A Belgian study demonstrated that a 10% reduction in PM_10_, PM_2.5_, and NO_2_ could result in substantial annual savings for cardiovascular hospitalisation costs, with 3.7 million, 3.6 million, and 5.9 million euros for arrhythmias, respectively, and 5.2 million, 5.0 million, and 8.4 million euros for ischemic heart diseases, respectively [12].

The implementation of air quality mitigation strategies can have various goals and additional consequences, such as complying with air quality standards, enhancing traffic flow, or addressing public health concerns [4]. Due to these abatement measures, air pollution concentrations have shown a decreasing trend, including the mortality rate associated with it [2,9]. As these strategies incur costs and generate benefits, economic evidence of these interventions can serve various purposes, including advocacy, resource allocation decisions, and policy-making decisions [13,14].

The cost-effectiveness of air pollution mitigation strategies has been reviewed in a few reviews, each with different inclusion criteria. A recent review on health economic analyses of air pollution control policies in China reported a wide variety of ways to analyse and report methods of cost-effectiveness of air pollution mitigation [15]. Another review summarised the health economic evidence in three environmental health areas, including air pollution. This study only included articles from 1990 until 2008 and concluded that the available health economic evidence was limited due to the small number of studies and variation in scientific quality and location [13]. These reviews did not assess the reporting quality of the included studies in a standardised way. A third more recent review only analysed cost–benefit analyses that included health benefits of air pollution mitigation strategies worldwide and divided these into groups (ambient, indoor, and mixed strategies). This article has a broad focus on the economic impact of air pollution control strategies worldwide. It assesses the reporting quality of the studies and stresses the need for more economic and epidemiological data to enable more robust health economic evaluations [16].

A significant gap has been identified in the literature regarding a comprehensive evaluation of cost–utility, cost-effectiveness, cost–benefit, and cost–minimisation analyses of air pollution mitigating strategies across various sectors. This review aims to address this gap by incorporating a broader range of economic evaluation methodologies, offering a more nuanced understanding of the financial implications and health benefits associated with air pollution mitigation strategies.

Specific focus will be placed on the potential cost-effectiveness of air pollution mitigation strategies, examining various interventions across different sectors. Additionally, this review will assess the reporting quality by means of the CHEERS II checklist, to enhance transparency and reliability in the presented findings.

This systematic review has the following objectives: (1) to investigate the published scientific evidence on the cost-effectiveness of strategies aimed at reducing or controlling air pollution and (2) to assess the reporting quality of the studies included in the literature review. As health economics and environmental health are both highly evolving fields, an up-to-date review may be useful in providing future researchers and policy maker with the latest relevant information. This review can contribute to essential insights for policy making, cost savings, and improvements to health.

## 2. Materials and Methods

A systematic review was conducted on the cost-effectiveness of air pollution interventions to reduce or control air pollution. This systematic review is performed according to the principles of the PRISMA guidelines, stated in Page et al. (2021) [17], and additional guidance was sought from established frameworks for conducting systematic reviews on the cost-effectiveness of health interventions [18,19,20]. A detailed protocol, developed in collaboration with another expert (NV), outlines the methodology for this systematic review. This document can be requested on demand. The study protocol was prospectively registered on PROSPERO (ID: CRD42023488753). There were no deviations from this protocol in this study.

### 2.1. Literature Search

Three databases have been systematically searched on the subject of cost-effectiveness of air pollution mitigation strategies. A search strategy has been developed for the database of MEDLINE (via PubMed) and has been adapted to EMBASE and Web of Science Core Collection (via Web of Science) (see Supplementary Materials). The grey literature has also been systematically searched for by means of Google (scholar) based on the following key terms mentioned below. Only policy reports have been included in the review for reasons of validity.

The search strategy is composed of the key concepts ‘air pollution’, ‘intervention(s) or strategy’, and ‘full health economic evaluations’. The concept ‘full health economic evaluations’ has been elaborated in another systematic review on the cost-effectiveness of the prevention of gestational diabetes, and these terms were also used in this review [21], with an update on a recent new MeSH term ‘cost-effectiveness’. The Boolean operators ‘AND’ and ‘OR’ were used to create a search strategy. This search strategy has further been reviewed by a librarian of the VUB University Medical Library. The last search was performed on 8 April 2025.

### 2.2. Study Selection

Eligibility criteria were defined using the PICO (Population, Intervention, Comparator, Outcome) strategy (Table 1). Exposure to the general population was included because air pollution is a ubiquitous environmental issue that affects all individuals, irrespective of demographic, geographic, or socioeconomic factors. Studies that focus on specific occupational exposures to certain types of air pollution were excluded. For example, the study of Ge et al. (2022) on inhalation of coal mine dust by coal workers or the study of Huang et al. (2021) on the exposure of aromatic hydrocarbons by Chinese chefs were excluded in this review [22,23]. This paper focuses on air pollution control strategies, both indoors and outdoors. It focuses exclusively on interventions that directly lead to air pollution reduction. Strategies or interventions where air pollution reduction is merely a co-benefit of other measures, such as climate mitigation, were excluded. Papers analysing the cost-effectiveness, cost–utility, or cost–benefit of air pollution abatement measures were eligible for inclusion, provided they described and compared the incremental costs and effects of two or more alternatives. Cost-minimisation analyses were also included. Economic evaluations that did not include health effects were excluded from this study. There were no restrictions on how health effects were expressed. Other systematic reviews or meta-analyses were excluded. No geographical restrictions were applied, and only articles written in Dutch, English, and French were included. Only articles published from 2013 onwards were eligible for this systematic review (as the cut-off was set at 10 years prior to the start of the screening process in October 2023).

For the screening process, the web application Rayyan was used [24]. All three reviewers (BV, LC, and NV) were blinded from each other’s decisions. One reviewer (BV) screened all records, while the other two reviewers each screened half of the articles. The first round screened the titles and abstracts. Next, all records that were retained from the first round were screened on full text, and reasons for exclusion were registered. In case of conflicts, a fourth independent reviewer (KP) acted as a tiebreaker. When full articles were not available, the original author was contacted via email. In addition, a ‘backward and forward citation’ search was performed to search for papers that have been overlooked.

### 2.3. Data Extraction

For data extraction from the papers included in this systematic review, a data extraction table was developed in Microsoft^®^ Excel based on an existing template [19]. The following characteristics were retrieved from the articles: (1) first author, (2) publication year, (3) country, (4) type of study, (5) intervention, (6) comparator, (7) sort model, (8) perspective, (9) time horizon, (10) costs, (11) effects/outcome, (12) type of sensitivity analysis, (13) result of sensitivity analysis, (14) incremental costs, (15) incremental effects, (16) ICER/ICUR,NB,CBR, (17) main conclusion, and (18) discount rate. One reviewer (BV) filled in this table, and when in doubt, she consulted a second independent reviewer (NV). The data extraction table has been validated by an expert (SS).

### 2.4. Synthesis of the Results

The results are summarised in evidence tables and figures or explained comprehensively in the main text. Because the articles included in this systematic review are difficult to compare, due to different methodologies and modelling, a narrative synthesis of the results is sufficient [25]. The grey literature included in this review is, together with the included scientific studies, referred as ‘articles’ in the Results section.

Guerriero et al. (2020) [26] state that the costs of a cost–benefit analysis can be divided into three major groups: compliance costs (purchase, construction, and maintenance); regulatory costs (government costs to implement the measure); and damage costs (e.g., environmental damage resulting from the intervention). However, they note that there are no clear specific guidelines for estimating the costs of an environmental intervention. Depending on the perspective, the type of intervention, and the number of markets affected, this cost analysis can vary [26]. We used the three abovementioned groups to analyse the costs from the articles. The benefits and effects of the interventions were also categorised to facilitate reporting [4,16]. Where possible, costs were converted to euros in the year 2024 using the online CCEMG–EPPI Centre Cost Converter [27].

### 2.5. Quality Assessment

Complete and transparent reporting of how health economic evaluation studies are designed and conducted is important to assess the validity of research findings and conclusions [28]. This is why the quality of reporting of the included articles was evaluated by means of the Consolidated Health Economic Evaluation Reporting Standards (CHEERS) II checklist. This is a guideline on the comprehensive and transparent reporting of health economic evaluations in the literature, to make these identifiable and useful in decision making. This 28-item checklist was used to assess the included papers [29]. This was performed by one reviewer (BV) and discussed with a second independent reviewer (NV). The results of this quality assessment were discussed narratively at the individual study level. An item was given a ‘1’ when reported in the article, ‘0’ when not reported, and ‘0.5’ when the reported information did not match entirely with the item. ‘NA’ was indicated when an item was not applicable for that particular study.

## 3. Results

### 3.1. Study Selection

**Figure 1:**
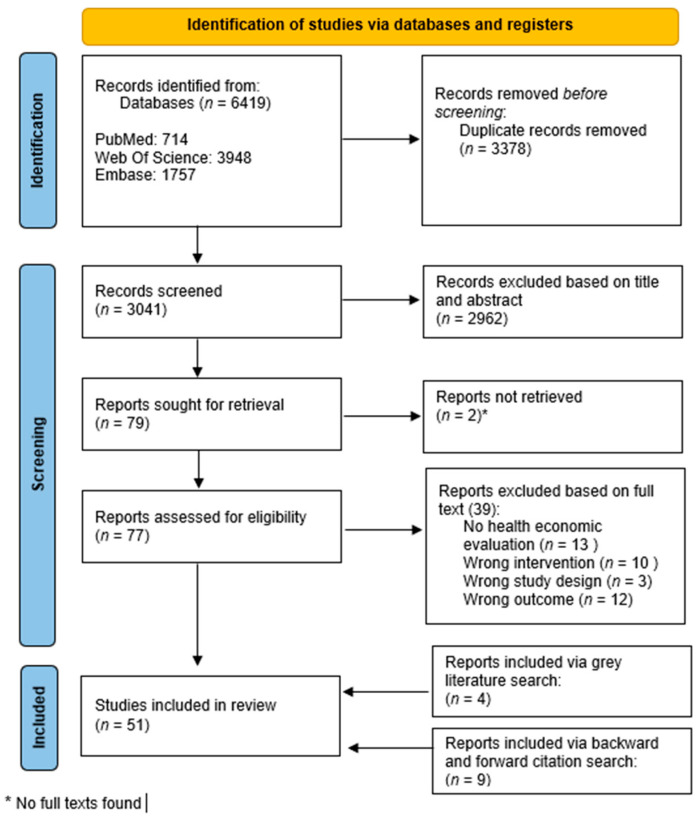
PRISMA flowchart of the literature search.

A total of 6419 articles were extracted from the aforementioned databases using the established search string (Figure 1). A large number of duplicates were removed (*n* = 3378), leaving 3041 articles to be screened based on their titles and abstracts. Of these, 2962 articles were excluded. Seventy-nine articles were subsequently screened based on the full text, with the full text of two articles not found. Attempts to contact the original authors were made, but no response was received. Of the 77 articles, 39 were excluded based on an intervention that did not meet the inclusion criteria (*n* = 10); a different study design than the inclusion criteria (*n* = 3); a different outcome than the inclusion criteria (*n* = 12); and no health economic evaluation (*n* = 13). Additionally, a ‘forward and backward citation search’ was conducted, resulting in nine additional articles being included. Finally, the grey literature was also searched, resulting in four additional reports being included. This makes a total of 51 studies retained for data extraction.

### 3.2. Study Identification

Of the 47 included studies, 40 report a cost–benefit analysis (CBA) [30,31,32,33,34,35,36,37,38,39,40,41,42,43,44,45,46,47,48,49,50,51,52,53,54,55,56,57,58,59,60,61,62,63,64,65,66,67,68,69] and 4 articles are cost–utility analyses (CUA) [14,70,71,72]. In three articles, it is not explicitly stated which analysis was performed, but it can be inferred that a cost–benefit analysis was conducted [73,74,75]. One policy report conducted both a CUA and a CBA [76], while the other three policy reports only performed a CBA [77,78,79]. In Table 2, the main characteristics of the studies are presented. Nineteen articles focus on China [34,36,37,38,41,44,47,49,54,56,57,58,59,61,64,67,68,75]; eight articles on Europe [30,35,48,50,51,53,71,74]; six on the United States [42,43,46,52,55,73]; two articles on the United Kingdom [14,72]; and eleven articles on other parts of the world such as Asia, Turkey, Canada, and South America [31,32,33,39,40,45,62,63,65,66,69,70]. Three of the four reports included in this review were set in the United Kingdom [76] and Europe [77,78], and one report is from India [79].

Since air pollution is a broad concept, the selected articles focused on a variety of pollutants. Particulate matter (PM_2.5_ of PM_10_) was investigated in 39 studies [14,31,32,33,34,35,36,38,39,40,41,42,43,46,47,48,49,50,51,52,54,56,58,59,60,61,62,64,65,66,67,68,70,71,72,77,78], and nitrogen oxides (NOx) were studied in 11 articles [14,44,48,50,51,54,55,57,59,67,79]. Ammonia (NH_3_), sulphur dioxide (SO_2_), and ozone (O_3_) were analysed in eight, nine, and nine articles, respectively (NH_3_ [30,35,37,53,74,75]; SO_2_ [44,54,55,57,59,63,67,79]; O_3_ [39,51,52,59,64,65,68,77]). Carbon (di)oxide (CO_2_) was reported in three articles [48,59,73]. Two studies did not specify which pollutant was addressed, simply referred to as ‘indoor air pollution’ [45,69]. Twenty-eight of forty-four articles analysed only one pollutant [30,31,32,33,34,36,37,38,40,41,42,43,46,47,49,53,56,58,60,61,62,66,70,71,72,73,74,75], while the other nineteen articles included multiple pollutants in their analyses [14,35,39,44,45,48,50,51,52,54,55,57,59,63,64,65,67,68,69].

The types of intervention discussed in the articles were divided into five groups: residential interventions (*n* = 15) [32,33,34,36,38,41,42,43,45,47,49,56,62,69,70]; industrial interventions (*n* = 12) [39,44,46,52,54,55,57,58,63,64,67,73]; transport interventions (*n* = 7) [14,40,48,60,61,66,76]; agricultural interventions (*n* = 6) [30,35,37,53,74,75]; and multiple interventions (i.e., spread across different sectors) (*n* = 5) [50,51,59,65,77]. In seven articles, no specific intervention was discussed [31,68,71,72,77,78,79]. These concern a reduction in the amount of a specific pollutant/emissions. An overview of the interventions per study can be found in Appendix A.

### 3.3. Characteristics of Health Economic Evaluations

Table 3 and Table 4 present the characteristics of the economic evaluations analysed in the included studies. Thirty-five articles do not report a clear perspective [30,31,33,34,36,37,38,39,40,42,43,45,46,47,48,49,50,51,52,54,55,56,57,59,62,63,64,65,67,71,72,73,75,78,79]. Seven CBA’s mention a societal perspective [44,58,60,61,68,74], and two report from a (local) government perspective [66,76]. In five CBAs, multiple perspectives are mentioned, encompassing both societal and individual perspectives (farmers, residents, and households) [32,35,41,53,69]. In two of the four CUAs, a healthcare perspective was clearly reported [14,70], while in the other two, no perspective was mentioned [71,72]. In 19 articles no time horizon was mentioned [30,31,33,35,36,38,39,40,42,47,48,49,50,51,56,62,73,74,75], but in 13 studies, it was explicitly mentioned in the methodology section [14,55,66,67,68,69,71,72,76,77,78,79].

According to Liu et al. (2023), CBAs can be categorised into two major groups: the traditional ex post evaluations and the ex ante simulations, also known as ‘integrated assessment models’ (IAMs) [15]. The first group evaluates already implemented policy measures, while the second group assesses potential future measures and predicts their impacts through modelling. This is achieved by combining various models. Most reported CBAs belong to the latter category (*n* = 31) [30,31,34,35,37,39,46,47,48,49,50,51,52,53,54,55,60,61,63,64,65,67,68,69,73,74,75,76,77,78,79]. Five articles are ex post CBAs [33,41,58,59,62]. In 11 articles, the methodology used was not specified [32,36,38,40,42,43,44,45,56,57,66]. Three CUAs utilised a Markov model [70,71,72], and in one article, the model applied was not specified [14].

In most CBAs, compliance costs are described (*n* = 27) [31,32,34,36,37,38,40,42,43,45,46,47,49,50,55,56,57,62,63,64,66,68,69,76,79]. Three articles incorporate these costs along with regulatory expenses in the analysis [33,41,67], while one article focuses solely on regulatory costs [52]. Three CBAs include additional costs, such as those related to accidents and noise; a reduction in gross profit margin due to mitigation measures; or the remaining expected value of ‘scrapped’ vehicles [48,53,60]. Among the four CUAs, three address healthcare costs [70,71,72], and in one article, this was not specified [14]. Two articles also included productivity loss as an indirect cost [77,78].

Each study examines health benefits based on various health outcomes. The most common are premature mortality and hospitalisations for various conditions such as lung diseases and cardiovascular diseases. In addition to health effects, six articles also consider productivity gains from reduced air pollution as benefits [41,45,51,58,59,60]. Seventeen other articles describe other benefits such as environmental, social, or economic benefits [32,34,35,37,44,45,48,55,58,60,61,62,64,65,66,69]. In 28 articles, the (health) outcomes are monetised using the Value of Statistical Life (VSL) [30,31,32,33,34,35,37,38,39,40,45,47,51,54,68,69,74,76,77,78,79] or by means of a willingness-to-pay (WTP) value (*n* = 3) [44,53,58]. Productivity gains are the most often monetised using the human capital method (*n* = 5) [44,56,60,61,62]. Ten other articles did not provide information on this aspect [36,41,42,43,46,49,50,55,73,75]. In the four CUAs, QALYs were evaluated [14,70,71,72].

In terms of discounting, both costs and effects should be discounted in economic evaluations to account for the time value of money, allowing them to be compared to each other. This way, a picture of the actual economic impact of a policy measure can be drawn. However, not every article appears to do this. In 29 articles, no discounting seemed to be applied at all [30,31,35,36,41,42,43,47,48,49,50,51,52,54,56,57,58,59,62,64,65,67,68,69,73,74,75]. In nine articles, only discounting of costs is mentioned, with rates ranging from 1.5% to 15% [32,33,34,37,39,40,44,53,66]. Thirteen articles discount both costs and effects, with a range of 1.5% to 12% [14,38,45,46,55,60,61,63,70,71,72].

Sensitivity analyses are an important component of economic evaluations. They map out the uncertainty of the data used. In 20 articles, no sensitivity analysis was shown or mentioned [14,30,39,43,46,50,51,54,58,59,60,61,62,65,67,68,69,73,75,79]. Six articles mention performing a sensitivity analysis but do not report the results [35,37,40,41,44,45]. Twelve articles report Monte Carlo simulations [32,33,34,36,38,47,49,52,56,57,71,72]. Eight studies conduct one-way sensitivity analyses [32,38,63,64,66,70,71]. Five articles report scenario analyses or probabilistic sensitivity analyses [31,42,48,53,74].

### 3.4. Results of Health Economic Evaluations

Table 5 and Table 6 show the results, outcome valuation and the sensitivity analysis results of the included studies.

The incremental costs and effects represent the difference in costs and effects between at least two interventions or strategies being compared. Only three articles explicitly mention incremental costs: one CBA and two CUAs [32,65,66]. Reporting on incremental benefits also varies, with only four articles explicitly mentioning them: two CBAs and two CUAs [32,34,65,66].

Eleven articles mention one or more non-cost-effective results [32,33,45,47,48,50,51,65,67,68,70], while the rest of the articles report only cost-effective results [30,31,34,35,36,37,38,39,40,41,42,43,44,46,49,52,53,54,55,56,57,58,59,60,61,62,63,64,66,69,71,72,73,74,75]. In four articles, no incremental cost–utility ratio (ICUR), cost–benefit ratio (CBR), or net benefits (NB) were mentioned [37,43,55,72]. In two cost–utility studies, an ICUR was calculated. One article analysed the different governmental reimbursement scenarios of air purifiers in various Canadian cities compared to full reimbursement and showed that only in one city would the base scenario be cost-effective (USD 38,628/QALY (EUR 31,878/QALY (2024)) at a WTP of USD 50,000/QALY (EUR 34,358/QALY (2024))) [70]. The other study analysed the implementation of US emission standards in Italy and France compared to the current national emission standards, reporting cost savings of, respectively, EUR 3000 (EUR 3952.78 (2024)) and EUR 1000 (EUR 1317.59 (2024)) and health gains of 0.31 and 0.04 QALYs [71].

Across various studies, similar interventions are often described, and in the majority of cases, the results are consistent (i.e., whether or not they are cost-effective). One exception is a residential intervention: the implementation of a liquefied petroleum gas (LPG) stove. Jeuland et al. (2016) [32] compare this intervention with standard wood-burning stoves and concluded it to not be cost-effective. Ramirez et al. (2024) [69] also find this to be the least cost-effective solution, compared with traditional biomass cooking stoves. In contrast, Irfan et al. (2016) identify it as the most cost-effective option, even in the most pessimistic scenarios, but they do not specify the comparative situation in their analysis. They report that in the most pessimistic scenario, all CBRs calculated, including those for LPG stoves, remain above 1, except for improved cookstoves (ICS). Jeuland et al. (2016) also shows a low probability of cost-effectiveness for ICS using charcoal or biomass (40% and 50%, respectively) [32,45]. Another negative outcome is reported by Schucht et al. (2018) and Miranda et al. (2016), who analyse different measures such as the implementation of hybrid and electric vehicles. They compare these scenarios with reference scenarios from, respectively, 2020 and 2012, which are not further specified. The intervention is not cost-effective, with net benefits of, respectively, −EUR 569 million (−EUR 719,277,863 (2024)) and −EUR 0.5 million (−EUR 867,685 (2024)) per year [50,51].

Wagner et al. (2015) [35], Giannadaki et al. (2019) [74], and Giannakis et al. (2019) [30] investigate the same agricultural interventions, including the use of low-nitrogen feed, manure storage capacity, low-emission animal housing, and the improvement or replacement of fertilisers. Wagner et al. (2015) [35] compare these interventions with the estimated emissions under current conditions, while Giannadaki et al. (2019) [74] and Giannakis et al. (2019) [30] do so using a control simulation, the details of which are not specified. All these studies report positive results: Giannakis et al. (2019) [30] and Wagner et al. (2015) [35] present these in the form of a cost–benefit ratio, while Giannadaki et al. (2019) [74] report net benefits [28,33,69]. However, due to the different comparators, no overarching conclusions can be drawn.

Other studies have shown that replacing traditional heating and cooking with solid fuels with gas and electricity is cost-effective [32,33,34,41,67], as is the use of solar panels [62]. The use of air purifiers in various forms has also been analysed in various articles. In all cases, air purifiers were found to be cost-effective [36,42,43,47,56,62,70], although the comparative situations varied: Fisk et al. (2017) [43], Fisk et al. (2017a) [42], Liu Y. et al. (2021) [47], and Yang et al. (2024) [36] compared the intervention to a baseline scenario without air purifiers, while Cansino et al. (2019) [62] compared it to the current situation involving wood-burning stoves. This makes it difficult to draw definitive conclusions. Lomas et al. (2016) [14] and Miranda et al. (2016) discuss the cost-effectiveness of implementing a low-emission zone. Both compare this measure to a baseline scenario from 2012 and report limited cost-effectiveness, depending on certain conditions [14,50].

The parameters that most influence cost-effectiveness results are discount rate [33,57,63], health-related parameters (dose–response coefficients, relative risk of asthma incidence, baseline prevalence of various conditions, and total mortality due to PM_2.5_) [33,38,57,71] and the value of VSL [38,57,63,66].

### 3.5. Results Quality Assessment

Each study was rated based on its quality of reporting using the CHEERS II checklist (Appendix B). Overall, the cost–benefit analyses seem to score relatively low (ranging from 12.5/28 to 21/28). Very often, no time horizon, perspective, or discount rate is described. Six CBAs do include these [32,44,53,60,61,66]. Policy reports also failed to include crucial elements of methodological reporting, scoring from 15.5 to 19 out of 28. The CUAs generally score higher (17/28 to 22/28). Items 18 (characterisation of heterogeneity), 19 (characterisation of distributional effects), 21 (approach to involving patients and others affected by the research), and 25 (impact of involving patients and others affected by the research) are less relevant to the topic of air pollution, so no article scored on these topics. The last two items have only recently been added to the CHEERS II checklist in 2022, making this result logical. Three articles scored on item 5 (population characteristics) [38,70,72]. All articles provided sufficient (albeit sometimes vague) information on item 6 (context and location) and item 7 (comparators). Only one article reports a health economic plan [70]. Item 27 (source of funding) was mentioned in 28 articles and 3 policy reports [14,30,38,41,42,43,44,46,47,48,49,50,51,52,53,54,55,56,57,59,60,61,62,66,67,68,71,75,76,78,79], and item 28 (conflict of interest) was mentioned in 26 articles [14,30,33,34,36,37,41,42,43,47,48,49,51,54,56,59,61,62,64,65,66,67,68,69,71,74].

## 4. Discussion

**Figure 2:**
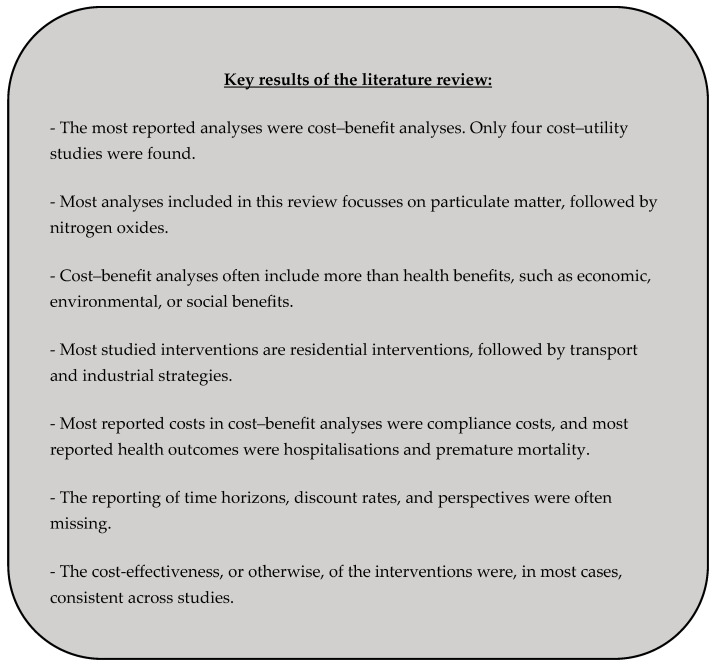
Key results of the literature review.

The aim of this systematic review was to inventory the published evidence on the cost-effectiveness of strategies to reduce air pollution. The key results of this literature review are shown in Figure 2. Most of the studies reviewed conducted a cost–benefit analysis (n = 48). This approach can be explained by the fact that air pollution is often evaluated more broadly than just from a health perspective [16,81]. Some studies also consider environmental, social, and economic benefits. Unlike cost-effectiveness or cost–utility studies, cost–benefit analyses encompass more than just health outcomes. This broader scope allows for cross-sectoral comparisons, which is reflected in several articles [82,83]. However, CBAs have their limitations. One of the main issues here is the diversity of monetisation approaches (VSL, WTP, and human capital approach) and the fact that there is no universal standard for valuing health effects. This lack of standardisation poses a significant shortcoming, making it challenging to compare different analyses [83].

Only a limited number of articles focus specifically on a cost–utility analysis. This indicates that little research has been conducted in this area, even though such an analysis could be particularly valuable for healthcare policy makers. A CUA can specifically emphasise health outcomes and quality of life. This allows for weighing the costs of the intervention against the health gains achieved [72]. Additionally, health outcomes expressed in generic outcome measures can be compared with other health interventions. When the primary goal of the analysis is to improve health by reducing air pollution, a cost-effectiveness or cost–utility study can be conducted [14]. However, if one opts for a cost–benefit analysis, the outcomes can still be monetised by, for example, using the willingness-to-pay (WTP) method [14,72,82].

The interventions in the articles are classified into four major groups according to Burns et al. (2020) [4]. A significant portion of the studies focus on residential interventions, emphasising indoor air pollution, in particular fine particulate matter (PM_2.5_ or PM_10_). Given that people spend up to 90% of their time indoors, these analyses are of great importance [84]. Most articles focus on low-income countries or poorer regions of countries like China, where households often still use solid fuels such as wood or coal for heating and cooking. In this context, new methods are introduced. These include the use of electricity and gas. The literature also indicates that the health effects of indoor air pollution in these countries are the most significant, as indoor air quality often exceeds recommended limits [85,86,87]. Almost all of these residual interventions are reported to be cost-effective. However, indoor air pollution in high-income countries also deserves attention. Urban areas often have high concentrations of ambient pollution, which can infiltrate indoors through ventilation systems and negatively impact health [84,88]. In this regard, six articles analyse the use of air purification devices in the home. This intervention is found to be cost-effective when compared with different scenarios. In urban areas, transport and industrial air pollution constitute the largest share of ambient air pollution. This is reflected by the fact that transport and industrial interventions are the second and third most reported intervention types in this review [87,89].

Air pollution is a broad concept, and the included studies focus on different types of pollutants. Agricultural interventions lead to a reduction in ammonia; traffic interventions reduce particulate matter (PM_2.5_ and PM_10_); and industrial interventions often focus on reducing SO_x_ and NO_x_, and other pollutants. Most articles focus on particulate matter, which has the largest share in health damage and causes millions of deaths worldwide annually [90]. Sixteen studies focus on multiple types of pollutants, named as multipollutant analyses. Liu, X. et al. (2023) mention that these analyses have become increasingly popular in China [15]. Since air pollution is an overarching term for various pollutants, all of which are present in the air simultaneously, it is sometimes difficult to study the effects of a single type of pollutant in isolation. An intervention or strategy will often reduce the emissions of multiple substances, making it more evident to apply a multipollutant analysis. Advantages of this approach include savings in time and money by reducing various pollutants together [91]. In a case study in Detroit, a multi-pollutant approach was compared with an approach where PM_2.5_ and O_3_ were measured and analysed separately. The results showed that a multipollutant approach led to a comparable or even greater reduction in both pollutants, resulting in improved quality of life. This approach also yielded twice the monetised benefits for PM_2.5_ and O_3_, reduced non-cancer risk, and proved to be more cost-effective than addressing both pollutants separately [92].

The methodologies of the included studies appear to be very different, making cross-comparison of the results challenging. The studies were conducted in both developed and developing countries, leading to significant differences in results. This difference is reflected in the valuation of benefits, which are sometimes calculated using WTP. These values are based on individual preferences and influenced by cultural norms, gender, age, and level of education, resulting in considerable international differences. To address this issue, organisations like the Organisation for Economic Co-operation and Development (OECD) have been generating reliable country-specific estimates of VSL [93]. Notably, 28 studies have utilised this measure, ensuring a more standardised approach to benefit valuation.

Additionally, perspectives often vary, meaning that different costs and effects are taken into account. This can influence the final results for cost-effectiveness, as also mentioned in Kim et al. (2020) [94]. Sometimes, the stated perspective does not match the included costs; for example, Giannadaki et al. (2018) only includes compliance costs from a societal perspective, while healthcare costs, which are normally included in such a perspective, are missing [74].

The time horizon chosen for a study significantly impacts its outcomes. Interestingly, this is mentioned in only a few studies and rarely in cost–benefit analyses. The studies included here often use a short time horizon of 5–10 years. However, air pollution reduction strategies typically incur high initial costs and only yield health benefits in the long term. Therefore, a sufficiently long time horizon is essential to fully capture their impact [80,95]. Discounting is another important aspect of health economic evaluations. Only 13 articles discount both costs and effects. Failing to discount effects can lead to an overestimation of future benefits, affecting the cost-effectiveness ratio and having significant policy implications [96]. It is crucial for studies to apply this methodology to provide a more accurate assessment of the true value of health interventions.

In addition to discounting costs and effects, conducting a sensitivity analysis is an essential part of health economic evaluations to test the robustness of the results. Only 17 articles performed a sensitivity analysis. This aligns with the findings of Jain et al. (2012) regarding other systematic literature reviews [97]. Moreover, there is a great variety in how these sensitivity analyses are conducted, which may be due to the lack of clear guidelines. This can complicate the interpretation and comparison of the results.

The previously mentioned shortcomings are also reflected in the quality assessment results. CBAs score significantly lower than CUAs. Only seven articles and one policy report mention the perspectives, time horizons, and discount rates, three important factors that, as previously indicated, influence the outcome of a cost-effectiveness study [98]. Only one article discusses a health economic protocol, which is understandable since this was only included in the CHEERS II checklist from 2022 [29]. The quality assessment of the included studies indicates that the reporting is generally of moderate quality. This lack of transparency hinders accurate interpretation of the results and makes it challenging to adequately assess the methodological robustness of the economic evaluations. Consequently, the reproducibility of these analyses is low.

All the diverse methodologies used in these studies make it challenging to draw a clear conclusion about the cost-effectiveness of these interventions. This difficulty is also highlighted by another systematic literature review that examined the health effects of air pollution reduction interventions. Burns et al. (2020) concluded that it was not possible to derive an overall conclusion of effectiveness due to heterogeneity of the included studies [4].

Additionally, the heterogeneity of the included articles makes the transferability of results to any specific country or region complex. Various contextual factors such as payment and reimbursement systems, economic climate, government and regulatory factors, and geographical factors can influence these decision-making processes [99]. Although models already exist that make this translation possible [100], these methods are complex and also have their limitations [101,102].

Upon reviewing the studies, three key recommendations emerged. Firstly, there is a critical need for financial support to implement environmental interventions in less developed countries and regions to achieve equitable implementation of, for example, air purifiers. This funding could be sourced from governments or other entities. Secondly, regional or national cooperation is essential for policy implementation, as air pollution transcends borders. For example, Gao et al. (2016) [44] lobby for increased communication and cooperation with other regions to encourage the development of the environmental technology industry. Lastly, increasing awareness among polluters, such as farmers and households, is crucial for gaining social acceptance and mitigating public resistance.

More research is needed on the cost-effectiveness or cost–utility of air pollution reduction from a healthcare perspective. Another future perspective for further research could be the importance of standardising methodologies for economic evaluations of environmental interventions and the need for long-term studies that capture the full impact of air pollution reduction strategies. The WHO published a report in 2000 about considerations in evaluating the cost-effectiveness of environmental health interventions [103]. Although this report may be outdated, it can still be used as a starting point for future economic evaluations. These studies should also report according to the CHEERS II checklist to make comparability possible.

Next to some opportunities for further research, this literature review also has several limitations. The first important limitation is the difficult delineation of inclusion criteria. Determining the centrality of air pollution proved to be challenging at times, potentially resulting in missing important studies. Another inclusion criterion was to include only articles from 2013 onward. As a result, we may have missed older relevant articles. Additionally, only articles written in English, Dutch, or French were included, meaning we might have missed studies in other languages. Another limitation of this paper concerns the categorisation of costs into specific sectors (such as the residential, transport, industrial, and agricultural sectors). This process was constrained by inconsistent reporting across the reviewed studies. As a result, we adopted broader cost groupings to ensure comparability and inclusiveness. This approach, while useful for identifying general trends, may limit the granularity of policy-specific insights that could be drawn from more sector-specific cost analyses. Furthermore, this paper did not differentiate findings based on the CHEERS II reporting scores of the included studies. While the CHEERS II checklist is a valuable tool for assessing the completeness of reporting in health economic evaluations, it does not directly reflect the methodological quality or validity of the evaluations themselves. This limitation restricts the ability to assess whether studies with higher reporting quality reach systematically different conclusions from those with lower reporting quality.

Lastly, ‘publication bias’ could result in fewer non-cost-effective interventions being reported compared to cost-effective interventions, which could have skewed the results of this review.

## 5. Conclusions

This systematic review fills a gap in the literature by examining the cost-effectiveness of environmental health interventions through various economic evaluation methodologies. It specifically highlights the potential cost-effectiveness of strategies aimed at mitigating air pollution. Overall, there is substantial evidence supporting the (non)-cost-effectiveness of interventions aimed at reducing air pollution, which is also stated in Wang et al. (2024) [16]. Predominantly, cost–benefit analyses have been reported, reflecting the multisectoral impacts and associated costs of air pollution, where cost–utility and cost-effectiveness analyses were less frequently documented. Across all studies, residential interventions were discussed the most, and significant attention was given to particulate matter and nitrogen oxides. The quality assessment revealed that the reporting quality, particularly of the cost–benefit analyses, is moderate. This heterogeneity is also reflected in other reviews on this topic [13,15,16].

This, combined with the heterogeneity of studies, makes it complicated to compare the results and form overall conclusions. By emphasising this difficulty, this review can contribute to a more standardised and robust framework for evaluating the economic impact and health benefits of environmental health interventions. The lack of cost–utility and cost-effectiveness studies indicate a need for further research on air pollution mitigation from a healthcare perspective, specifically through health economic evaluations. This can be invaluable for policy makers focusing on healthcare and public health.

The common thread in the policy recommendations that emerged from the studies proves that there is a consensus among researchers on the key strategies needed to effectively address environmental health issues. This alignment underscores the importance of financial support, regional cooperation, and increasing awareness among polluters as fundamental components of successful environmental interventions. It also highlights the need for a standardised approach to policy implementation, ensuring that efforts are coordinated and impactful across different regions and sectors.

## Figures and Tables

**Table 1:** Study eligibility criteria.

Parameter	Inclusion	Exclusion
**Population**	General exposure to air pollution	Occupational exposure
**Intervention**	Strategies aimed at reducing or controlling air pollution both indoors and outdoors.	Strategies where the reduction in air pollution occurs as a ‘co-benefit’
**Comparator**		/
**Outcome**	Studies that take costs and health outcomes into account to calculate ICER/ICUR/NB/CBR	Only costs, exclusion of health outcomes
**Study design**	Cost-effectiveness analysis, cost–utility analysis, cost–benefit analysis, cost-minimisation analysis, pre–post study designs, (non-)randomised controlled trials	Other study designs (i.e., reviews, meta-analyses)
**Geography**	No limitations	/
**Language**	English, French, Dutch	Other languages
**Publication date**	From 2013	Before 2013

ICER: incremental cost-effectiveness ratio; ICUR: incremental cost–utility ratio; NB: net benefit; CBR: cost–benefit ratio.

**Table 2:** Main characteristics of the included studies.

Cost–Benefit Analysis								
Author (Publication Year)	Country	Sort of Pollutant						
		NH_3_	PM *	NO_x_	O_3_	SO_2_	CO_x_	NS
**Aunan et al. (2013)** [38]	China		x					
**Cansino et al. (2019)** [62]	Temuco (Chile)		x					
**Chen et al. (2015)** [39]	East Asia		x		x			
**Cropper et al. (2017)** [63]	India		x			x		
**Evans et al. (2021)** [40]	Mexico City		x					
**Feng et al. (2021)** [41]	China		x					
**Fisk et al. (2017)** [43]	South California (USA)		x					
**Fisk et al. (2017a)** [42]	USA (Los Angeles CA, Elizabeth NJ, Houston TX)		x					
**Gao et al. (2016)** [44]	China		x	x		x		
**Giannakis et al. (2019)** [30]	Europe	x						
**Guo et al. (2023)** [64]	China		x		x			
**Howard et al. (2019)** [31]	Northeast Brazil		x					
**Irfan et al. (2021)** [45]	Pakistan							x
**Jeuland et al. (2016)** [32]	South Asia/global		x					
**Lavee et al. (2018)** [65]	Israël		x		x			
**Levy et al. (2017)** [46]	New York		x					
**Liu K et al. (2024)** [67]	China		x	x		x		
**Liu Y. et al. (2021)** [47]	China		x					
**Liu Z et al. (2023)** [68]	China		x		x			
**Lopez-Aparicio et al. (2020)** [48]	Oslo (Norway)		x	x			x	
**Mardones et al. (2021)** [33]	Southern Chile		x					
**Meng W. et al. (2023)** [34]	Jing-Jin-Ji Region (China)		x					
**Meng W. et al. (2023a)** [49]	China		x					
**Miranda et al. (2016)** [50]	Grande Porto area		x	x				
**Ramirez et al. (2024)** [69]	Nepal							x
**Schucht et al. (2018)** [51]	France		x	x	x			
**Thompson et al. (2016)** [52]	USA		x		x			
**Wagner et al. (2015)** [35]	Germany (German Federal States of Lower Saxony, Brandenburg, Baden-Württemberg)	x	x					
**Wagner et al. (2017)** [53]	Germany (Lower Saxony)	x						
**Wan et al. (2023)** [54]	China		x	x		x		
**Whitehurst et al. (2021)** [66]	Canada		x					
**Wiser et al. (2017)** [55]	US			x		x		
**Yang et al. (2024)** [36]	China (Beijing, Harbin, Shanghai, Guangzhou, Sanya, Kunming)		x					
**Zhang et al. (2015)** [57]	China			x		x		
**Zhang et al. (2019)** [58]	China		x					
**Zhang et al. (2020)** [37]	China	x						
**Zhang et al. (2023)** [56]	China		x					
**Zhao et al. (2022)** [59]	China		x	x	x	x	x	
**Zhou et al. (2019)** [60]	China (BTH region)		x					
**Zhou et al. (2022)** [61]	China (BTH region)		x					
**Cost–utility analysis**								
**Adibi et al. (2023)** [70]	Canada		x					
**Kim et al. (2020)** [71]	Italy, France		x					
**Lomas et al. (2016)** [14]	UK		x	x				
**Schmitt L.H.M. (2016)** [72]	UK		x					
**Not specified**								
**Buonocore et al. (2016)** [73]	USA						x	
**Giannadaki et al. (2018)** [74]	Europe, America, Asia	x						
**Liu M. et al. (2019)** [75]	China	x						

UK: United Kingdom; USA: United States of America; BTH region: Beijing–Tianjin–Hebei region; NS: not stated. *: PM_2.5_ and PM_10._

**Table 3:** Characteristics of the included health economic evaluations.

Author (Publication Year)	Study Design	Model	Perspective	Time Horizon	Costs	Valuta	Discount Rate	Reference Year
**Adibi et al. (2023)** [70]	CUA	Markov model	Healthcare perspective	2018–2022	Healthcare costs, Compliance costs	CAD	Costs: 1.5% Benefits: 1.5%	2023
**Aunan et al. (2013)** [38]	CBA	Not specified	/	/	Compliance costs	CNY	Costs: 8% Benefits: 8%	2010
**Buonocore et al. (2016)** [73]	Not specified	Combination of Integrated Planning Model + Community Multiscale Air Quality Model + BenMAP CE	/	/	Compliance costs	USD	/	2013
**Cansino et al. (2019)** [62]	CBA	Ex post evaluation	/	/	Compliance costs	USD	/	2013
**Chen et al. (2015)** [39]	CBA	Combination of CMAQ/REAS + GAINS + arc GIS system models	/	/	Not specified	USD	Costs: 10% Benefits: /	2020
**Cropper et al. (2017)** [63]	CBA	Combination of CAMx + IERs	/	/	Compliance costs	USD	Costs: 3% Benefits: 3–8%	2015
**Evans et al. (2021)** [40]	BKA	Not specified	/	/	Compliance costs	USD	Costs: 3% Benefits: /	/
**Feng et al. (2021)** [41]	CBA	Ex post evaluation (difference-in-difference modelling)	Costs: governmental perspective Benefits: residents	2015–2018	Compliance costs, Regulatory costs	CNY	/	/
**Fisk et al. (2017)** [43]	CBA	Not specified	/	/	Compliance costs	USD	/	2003
**Fisk et al. (2017a)** [42,43]	BCA	Not specified	/	/	Compliance costs	USD	/	/
**Gao et al. (2016)** [44]	CBA	Not specified	Societal perspective	5 years	Not specified	CNY	Costs: 3% Benefits: /	2012
**Giannadaki et al. (2018)** [74]	Not specified	Combination of ‘EMAC global atmospheric chemistry–climate model + health impact function + exposure response function’	Societal perspective	/	Compliance costs	USD	/	2010
**Giannakis et al. (2019)** [30]	CBA	Combination of models: WRF/Chem model	/	/	Not specified	M EUR	/	2016
**Guo et al. (2023)** [64]	CBA	Combination of WRF/Chem model + health impact assessment	/	/	Compliance costs	USD	/	2015
**Howard et al. (2019)** [31]	CBA	Combination of Plexos + CALPUFF + BenMAP models	/	/	Compliance costs	USD	/	2015
**Irfan et al. (2021)** [45]	CBA	Not specified	/	2014–2024	Compliance costs	INR, USD	Costs: 3%; 7.5%; 12% Benefits: 3%; 7.5%; 12%	2014
**Jeuland et al. (2016)** [32]	CBA	Not specified	Household and societal perspective	100	Compliance costs	USD	Private costs: 5–15% Social costs: 1–6%	/
**Kim et al. (2020)** [71]	CUA	Markov model	/	Life time	Compliance costs, Healthcare costs	EUR	Costs: 3% QALYs: 3%	2018
**Lavee et al. (2018)** [65]	CBA	Combination of ‘IMoEP air quality forecast models + dose–response functions’	/	/	Not specified	ILS	/	/
**Levy et al. (2017)** [46]	CBA	Combination of AERMOD + BenMAP models	/	20 years	Compliance costs	USD	Costs: 3% Benefits: 3%	/
**Liu K et al. (2024)** [67]	CBA	Combination of models: Facility-level emission inventory + CMAQ + GEMM	/	2020–2060	Compliance costs Regulatory costs	CNY	/	2020
**Liu M. et al. (2019)** [75]	Not specified	Combination of WRF/Chem + GAINS model	/	/	Not specified	USD	/	/
**Liu Y. et al. (2021)** [47]	CBA	Combination of exposure assessment model + health risk assessment model + cost-effectiveness assessment model	/	/	Compliance costs	CNY	/	/
**Liu Z et al. (2023)** [68]	CBA	Combination of DPEC model + RSM	Societal perspective	2020–2060	Compliance costs	USD	/	2020
**Lomas et al. (2016)** [14]	CUA	Not specified	Healthcare perspective	Life time	Not specified	GBP	Costs: 3.5% Benefits: 3.5%	/
**Lopez-Aparicio et al. (2020)** [48]	CBA	Combination of ‘emission inventory + different atmospheric dispersion models + population exposure’	/	/	Other costs: private and social costs, health, climate, accidents, noise	NOK	/	2019
**Mardones et al. (2021)** [33]	CBA	Ex post evaluation (difference-in-difference modelling)	/	/	Compliance costs, Regulatory costs	CLP	Costs: 6%	/
**Meng et al. (2023)** [34]	CBA	Combination of models: GAINS model	/	2020–2030	Compliance costs	CNY	Costs: 4%	2020
**Meng et al. (2023)** [49]	CBA	Combination of ‘Energy consumption and emission modelling + WRF/Chem model’	/	/	Compliance costs	RMB	Costs: 6% Benefits: /	2021
**Miranda et al. (2016)** [50]	CBA	Combination of models, not specified,	/	/	Compliance costs	M EUR	/	2012
**Ramirez et al. (2024)** [69]	CBA	Open-source clean cooking cost–benefit analysis tool OneStove + multicriteria analysis based on the Energy Access Explorer methods	Societal perspective Private household perspective	2021–2030	Compliance costs	USD	/	2021
**Schmitt L.H.M. (2016)** [72,80]	CUA	Markov model	/	60 years	Healthcare costs	GBP	Costs: 3.5% Benefits: 3.5%	2013
**Schucht et al. (2018)** [51]	CBA	Combination of CHIMERE ARP-France model	/	/	Not specified	M EUR	/	2012
**Thompson et al. (2016)** [52]	CBA	Combination of ‘United Stated Energy Policy + Comprehensive Air quality Model with Extensions + BenMAP + mortality incidence’	/	/	Regulatory costs	USD	/	2006
**Wagner et al. (2015)** [35]	CBA	Combination of EFEM + EcoSense modelling	Costs: farmer’s perspectiveBenefits: societal perspective	/	Not specified (‘reduction cost’)	M EUR	/	2015
**Wagner et al. (2017)** [53]	CBA	Combination of economic–ecological farm model + integrated environmental assessment model	Costs: farmer’s perspectiveBenefits: societal perspective	2015–2050	Other costs: reduction in gross profit margin due to mitigation measures	EUR	Costs: 3% in 2030 and 2% from 2030 to 2050 Benefits: /	2015
**Wan et al. (2023)** [54]	CBA	Combination of ‘China Emissions Accounts for Power Plants database + WRF-CAMx + IMED/HEL + LCOE model’	/	/	Not specified	CNY	/	2015
**Whitehurst et al. (2021)** [66]	CBA	Not specified	Perspective of local government	10 years	Compliance costs	USD	Costs: 1.5% Benefits: /	2016
**Wiser et al. (2017)** [55]	CBA	Combination of models: an electric generation capacity expansion model	/	2015–2050	Compliance costs	USD	Costs: 3% Benefits: 1.5%	/
**Yang et al. (2024)** [36]	CBA	Not specified	/	/	Compliance costs	CNY	/	/
**Zhang et al. (2015)** [57]	CBA	Not specified	/	2006–2015	Compliance costs	USD	/	2006
**Zhang et al. (2019)** [58]	CBA	Ex post evaluation	Societal perspective	/	Not specified	RMB	/	2013
**Zhang et al. (2020)** [37]	CBA	Combination of CHANS + GAINS + WRF-CMAQ + exposure response models	/	/	Compliance costs	CNY	Costs: 2%	2015
**Zhang et al. (2023)** [56]	CBA	Not specified	/	/	Compliance costs	CNY	/	/
**Zhao et al. (2022)** [59]	CBA	Ex post evaluation	/	2016–2018	Not specified	CNY	/	/
**Zhou et al. (2019)** [60]	CBA	Combination of WRF-CMAQ-response functions–economic evaluation model	Societal perspective (government residents, enterprises)	2008–2015	Other costs	CNY	Costs: 8% Benefits: 8%	2015
**Zhou et al. (2022)** [61]	CBA	Idem Zhou et al. (2019) [60]	Societal perspective	2008–2016	Not specified	CNY	Costs: 8% Benefits: 8%	2015

/: not reported; CBA: cost–benefit analysis; CUA: cost–utility analysis; EMAC: ECHAM/MESSy Atmospheric Chemistry; AERMOD: AMS/EPA Regulatory Mode; WRF/Chem: Weather Research and Forecasting Model with Chemistry module; CALPUFF: California Puff Model; BenMAP (-CE): Environmental Benefit Mapping and Analysis Program (–Community Edition); GAINS: Greenhouse Gas–Air pollution Interactions and Synergies; EFEM: Economic Farm Emission Model; CHIMERE ARP-France model: HIMistry and Emissions Research Alpha-RiskPollFrance; CHANS: Coupled Human And Natural Systems; WRF-CMAQ: Weather Research and Forecasting Model–Community Multiscale Air Quality Modeling System; WRF-CAMx: Weather Research and Forecasting-Comprehensive Air Quality Model with Extensions; IMED/HEL: the Integrated Model of Energy, Environment, and Economy for Sustainable Development/Health impact assessment; LCOE model: the levelised cost of electricity; CAMx: Comprehensive Air Quality Model with Extensions; CMAQ: Community Multiscale Air Quality; GEMM: Global Exposure Mortality Model; DPEC: Dynamic Projection Model for Emission in China; RSM: Response Service Models; IERs: Integrated Exposure Response functions; IMoEP: Integrated Model for the Optimal Estimation of Pollution; M EUR: million euros; USD: American dollars; CLP: Chilean pesos; CNY: Chinese yuan; NOK: Norwegian Kroner; RMB: Chinese renminbi; ILS: Israeli shekel; £GBP: British pound sterling; INR: India Rupee.

**Table 4:** Characteristics of the included grey literature.

Author (Publication Year)	Study Design	Model	Perspective	Time Horizon	Costs	Valuta	Discount Rate	Reference Year
**Amann et al. (2017)** [77]	CBA	Combination of models: GAINS	Societal perspective	2005–2030	Compliance costs, Regulatory costs	EUR	/	2005
**Ballinger et al. (2016)** [76]	CBA and CUA	Not specified	Perspective of local government	30 years	Compliance costs	GBP	Costs: 3.5% Benefits: 3.5%	/
**Holland et al. (2014)** [78]	CBA	Combination of models: GAINS	/	2010–2030	Healthcare costs (direct and indirect)	EUR	/	2005
**Srinivasan et al. (2018)** [79]	CBA	Combination of models: CAMx	/	2015–2030	Compliance costs	INR	Costs: 8% Benefits: 8%	2015

/: Not reported; CBA: cost–benefit analysis; CUA: cost–utility analysis; GAINS: Greenhouse Gas–Air pollution Interactions and Synergies; CAMx: Comprehensive Air Quality Model with Extensions; £GBP: British pound sterling.

**Table 5:** Outcome valuation, sensitivity analysis, and results of included health economic evaluations.

Author (Publication Year)	Outcome (Valuation Outcome)	Sensitivity Analysis	Incremental Costs (1) Incremental Benefits (2)	Cost-Effectiveness Results	Results of Sensitivity Analysis
**Adibi et al. (2023)** [70]	QALYs (EQ-5D)	OWSA, PSA	(1) USD 70.9–86.4 (2) 0.0018–0.0010 QALY	ICERs between USD 38,628 and 85,445 **(±)**	PSA: 80.2% in KB, 43.6% in Ok, 29.6% in TCS. OWSA: risk ratio of increased salbutamol dispensation and hospitalisation, utility of well-controlled and uncontrolled asthma, and retail price of air filter units are the most influential parameters.
**Aunan et al. (2013)** [38]	Health benefits (VSL)	OWSA Monte Carlo analysis	/	BCR: S1a: 14.7; S1b: 3.3, S2a: 14.5, S2b: 3.7 **(+)**	OWSA: lifetime of intervention, value of VSL, and baseline COPD prevalence are the most influential parameters.
**Buonocore et al. (2016)** [73]	Health benefits (/)	/	/	NB: −USD 2.3–1.7 billion **(+)**	/
**Cansino et al. (2019)** [62]	Health and social benefits: less accidents (VSL/HCM)	/	/	Benefits exceed costs (no numbers) **(+)**	/
**Chen et al. (2015)** [39]	Health benefits (VSL)	/	/	BCR: FS: 9.0–25; FR: 25–68 **(+)**	/
**Cropper et al. (2017)** [63]	Health benefits (VSL)	OWSA	/	CBR: 0.31–18 NB: −USD 95.7–2870 million **(+)**	The size of the present value of mortality benefits is sensitive to VSL and discount rate.
**Evans et al. (2021)** [40]	Health benefits (VSL)	PSA	/	NB: USD 150 million/year **(+)**	PSA: 88–97% prob. on cost-eff.
**Feng et al. (2021)** [41]	Health benefits (/)	Mentioned but not reported	/	NB: CNY 289.54–26,234.44 million CBR: 1/4.49 **(+)**	/
**Fisk et al. (2017)** [43]	Health benefits (/)	/	/	Intervention cost exceeds the economic benefits, but economic benefits of reduced mortality exceed the intervention costs of interventions i1–i3 **(+)**	/
**Fisk et al. (2017a)** [42,43]	Health benefits (/)	PSA	/	BCR: i1: circa 4. i2: 14–25, i4–i7: 6–13, i8–i9: 74–133 (i3, i8, i9 have the lowest cost/premature mortality) **(+)**	/
**Gao et al. (2016)** [44]	Health, climate, and economic benefits (HCM, WTP)	Performed but not specified	/	NB: CNY 629.76 billion BCR: 1.10–38.25 **(+)**	Unit emission reduction costs, unit subsidy, and GDP growth rate are the most sensitive in all scenarios.
**Giannadaki et al. (2018)** [74]	Health benefits (VSL)	Scenario analysis	/	Net economic benefits: (1) 87.9 (2) 65.0 (3) 84.3 (4) 163 (5) 85.3 **(+)**	/
**Giannakis et al. (2019)** [30]	Health benefits (VSL)	/	/	CBR: (1) 186 (2) 63 (3) 4 (4) 59 **(+)**	/
**Guo et al. (2023)** [64]	Health and climate benefits (VSL)	OWSA	/	NB: USD 30–156 billion **(+)**	/
**Howard et al. (2019)** [31]	Health benefits (VSL)	Scenario analysis	/	BCR: (1) 60 (2) 103 (3) 89 **(+)**	- Dry years: PM_10_ emissions under more stringent standards increase by 18.5%. - The use of an alternative concentration response function increases mortality by a factor of 2.9–4.9.
**Irfan et al. (2021)** [45]	Health, economic, and climate benefits (VSL)	Scenario analysis	/	BCR: 0.38–4.64 NPV:–PKR 338.161 for different measures **(±)**	Even in most pessimistic scenario, the BCR is above 1, except for ICS.
**Jeuland et al. (2016)** [32]	Health, private, and social benefits (COI, VSL)	Monte carlo analysis, OWSA	/	Household perspective: all except LPG give +NB, due to high fuel cost Social perspective: significant social benefits **(±)**	Probability of private (and social) benefits: LPG stoves: 37% (70%); Biomass ICS: 40% (30%); Charcoal ICS: 50% (70%); Electric ICS: 64% (30%). OWSA: time savings and fuel costs have the most impact on net benefits.
**Kim et al. (2020)** [71]	QALYs (utilities)	OWSA Monte Carlo analysis	(1) EUR 1000 (France); EUR 3000 (Italy) (2) 0.04 QALY (France) 0.31 QALY (Italy)	Dominant result (ICER not calculated) **(+)**	OWSA: relative risk of asthma incidence in France and continuous cost for chronic CVD in Italy. Monte Carlo: 93.8% (Fr), 87.4% (It) were cost- and life-saving; 0.7% (Fr) and 10.1% (It) fell below WTP EUR 46,000; 98.7% (Fr), 96.0% (It) prob of cost-eff. on CEAC with WTP EUR 46,000.
**Lavee et al. (2018)** [65]	Health and other benefits: improved safety, savings on fuel (VSL)	/	/	NB: −ILS 6.6–400 million for different measures **(±)**	/
**Levy et al. (2017)** [46]	Health benefits (VSL)	/	(1) USD 190,000 (annual) (2) −USD 1.7 million	BCR: 9.7 **(+)**	/
**Liu K et al. (2024)** [67]	Health benefits (/)	/	/	CBI (deaths/million CNY) (3): 2.9; (5): 4.6; (6): 1.4	/
**Liu M. et al. (2019)** [75]	Health benefits (/)	/	/	NB: USD 0.4 billion **(+)**	/
**Liu Y. et al. (2021)** [47]	Health benefits (VSL)	OWSA Monte Carlo analysis	/	NB: C-B: S1: 131; S2: 90; S3: −60; S4: −317 billion CBR: C/B per scenario: S1: 2.6; S2: 1.5; S3: 0.9; S4: 0.6 **(±)**	/
**Liu Z et al. (2023)** [68]	Health benefits (VSL)	/	/	CBR: NOx-AVOC-A: USD 0.23 trillion in BTH; USD 0.12 trillion in YRD (significant) NOx-AVOC-B: cost-effective regional but less nationwide NOx only: less effective	/
**Lomas et al. (2016)** [14]	QALYs (HRQoL)	/	/	/	/
**Lopez-Aparicio et al. (2020)** [48]	Health benefits (/)	PSA	/	BCR = 1.24 (Scenario 1); 0.79 (Scenario 2) **(±)**	Conservative or high estimates do not significantly alter the result, but varying the cost of time has the largest effect, with net changes to the results varying by a maximum of 20%.
**Mardones et al. (2021)** [33]	Health benefits (VSL)	Monte Carlo analysis	/	CE **(±)**: (1) CLP 5016/kg PM_2.5_; (2) CLP 5854/kg PM_2.5_; (4) ‘Infinite’ CBR: (1) 0.40; (2) 0.47; (4) 0	- The effects of the replacement program on emissions; - Discount rate; - Dose–response relationships are the most influential parameters.
**Meng et al. (2023)** [34]	Health and climate benefits (VSL)	Monte Carlo analysis	(1) (only in figure): 1. CNY ±6000; 2. ±5800; 3. ±5800; 4. ±1500; 5. ±2000 /household (2) 1, 2, 3. CNY 100; 4. 53; 5. 59 million	BCR **(+)** (Only reported in figure): 1. ±5 2. ±5 3. ±5 4. ±22 5. ±20	Sources of uncertainty: Costs: costs of power plants, power grids, natural gas pipelines, household appliances, and fuel Benefits: premature deaths, the value of a statistical life, and the social cost of carbon
**Meng et al. (2023)** [49]	Health benefits (/)	Monte Carlo (but no results reported) + SA on total cost	/	RMB 2.3 million /avoided death **(+)**	The gas price was the most significant factor that influenced the total cost.
**Miranda et al. (2016)** [50]	Not specified (/)	/	/	HYB: NB = −M EUR 0.5 /y; BKR = 0.75 FIR: NB = M EUR 1.0 /y; BKR = 2.25 LEZ: NB = M EUR 0.001 /y; BKR = 1.03 IND: NB = −M EUR 0.2 /y; BKR = 0.97 HYB + FIR: NB = M EUR 0.5 /y; BKR = 1.18 FIR + IND: NB = M EUR 0.9 /y; BKR = 1.14 HYB + FIR + LEZ + IND: NB = M EUR 0.3 /y; BKR = 1.03 **(±)**	/
**Ramirez et al. (2024)** [69]	Health, climate, and other benefits: time spent collecting fuel and cooking (VSL)	/	/	1. Electric Stove: 9563 deaths, USD 1.27B saved/y 2. LPG: 8758 deaths, USD 0.96B saved/y 3. Biogas: 9267 deaths, USD 1.02B saved/y 4. Improved Biomass Cookstoves: 2833 deaths, USD 0.31B saved/y	/
**Schmitt L.H.M. (2016)** [72,80]	QALYs (/)	PSA, Monte Carlo analysis	/	/	/
**Schucht et al. (2018)** [51]	Health benefits (/)	/	/	NB for 48 different measures, both (non-)cost-effective **(±)**	/
**Thompson et al. (2016)** [52]	Health benefits (VSL)	Monte Carlo analysis	/	NB not specified	/
**Wagner et al. (2015)** [35]	Health, climate, and environmental benefits (VSL)	Performed but not specified	/	BCR: (1) 8.1 (BW), 8.4 (Br) (2)/ (3) 0.8 (poultry), /(pigs) (4) 7.6 (gran), 2.4 (sw. foil), 5.2 (concr. cover) (5) 0.9 (tr. hose), 3.2 (tr. shoe), 3.9 (inject) (6) 4.8 (chem. was), 2.2 (3state-syst), /(biofilter) **(+)**	The net benefits and GHRs of most measures remained positive even with variations in model parameters (except for biofilters).
**Wagner et al. (2017)** [53]	Health benefits (WTP)	PSA	/	Fl. Shoe: NB: 505 million, BKR: 4.2 Conc Inject: NB: 401 million, BKR: 3.6 **(+)**	When varying abatement potential, abatement costs, and avoided damage costs, abatement measures were consistently cost-effective.
**Wan et al. (2023)** [54]	Health benefits (VSL)	/	/	Most control measures yield monetised net benefits (no figures) **(+)**	/
**Whitehurst et al. (2021)** [66]	Health and climate benefits (HEAT)	OWSA	/	CBR: 0.1–4.9 for the different cities **(+)**	Time horizon, investment cost, and VSL values are the most influential parameters.
**Wiser et al. (2017)** [55]	Health and climate benefits (/)	Not specified	/	/	Not specified
**Yang et al. (2024)** [36]	Health benefits (VSL)	Monte Carlo analysis	(1) / (2) incremental DALY, but no outcomes reported	Different NE and COE in cities of China (no figures but shown on diagram)	/
**Zhang et al. (2015)** [57]	Health benefits (VSL)	Monte Carlo analysis	/	Average net benefit is USD 53.2/MWh for 600 MW generated under multi-pollutant strategies, USD 6.5/MWh higher than graduated pollutant strategy **(+)**	Capital cost and O&M costs have a small influence; discount rate has the most influence on control costs, and the intake fraction of sulphates and nitrates; and the CRR for total mortality has the greatest influence on health benefits. Health benefits are most sensitive to VSL values.
**Zhang et al. (2019)** [58]	Health and environmental benefits (WTP)	/	/	NB: RMB 818 billion CBR: 1.49 **(+)**	/
**Zhang et al. (2020)** [37]	Health and climate benefits (VSL)	/	/	/	/
**Zhang et al. (2023)** [56]	Health benefits (HCM)	Monte Carlo analysis	/	NB **(+)**: S1: 184; S2: 275; S3: 301; S4: 203	/
**Zhao et al. (2022)** [59]	Health benefits (VSL)	/	/	Ratio of economic benefit to government expenditure: 63.7% **(+)**	/
**Zhou et al. (2019)** [60]	Health and environmental benefits (HCM, COI)	/	/	NB: CNY 20.34 billion CBR: 1:2.49 **(+)**	/
**Zhou et al. (2022)** [61]	Health and private benefits	/	/	NB: CNY 92.69 CBR: 1:16.97 **(+)**	/

/: not reported; BCR: benefit–cost ratio; BTH: Beijing–Tianjin–Hebei; Br: Brandenburg; BW: Baden-Württemberg; CE: cost-effectiveness; CEAC: cost-effectiveness acceptability curve; CNY: Chinese yuan; COE: coefficient of economic benefit; COI: cost of illness; COPD: chronic obstructive pulmonary disease; CRR: concentration–response rate; CVD: cardiovascular diseases; DALY: disability-adjusted life year; EQ-5D: EuroQol-5 Dimensions questionnaire; GDP: gross domestic product; HCM: human capital method; HEAT: health economic assessment tool; ICER: incremental cost-effectiveness ratio; ICS: improved cooking stove; LPG: liquid petroleum gas; NB: net benefit; NE: net economic benefit; ILS: Israeli shekel; NPV: net present value; OWSA: one-way sensitivity analysis; PKR: Pakistan rupee; PSA: probabilistic sensitivity analysis; QALY: quality-adjusted life year; RMB: renminbi; SA: sensitivity analysis; VSL: value of statistical life; WTP: willingness to pay; YRD: Yangtze River Delta. (+): only cost-effective results; (±): both cost-effective and non-cost-effective results.

**Table 6:** Results of included health economic evaluations from the grey literature.

Author (Publication Year)	Outcome (Valuation Outcome)	Sensitivity Analysis	Incremental Costs (1) Incremental Benefits (2)	Results	Results of Sensitivity Analysis
**Amann et al. (2017)** [77]	Health, climate, and other benefits (VOLY, VSL)	Monte Carlo analysis	(1) / (2) /	BCR > 14 for lower estimate of mortality BCR > 50 for the higher estimate (+)	Benefits of the actions identified using the GAINS model significantly exceed the costs even under conservative assumptions.
**Ballinger et al. (2016)** [76]	Health benefits (VSL)	PSA	(1) / (2) /	ICER: GBP 441–25.199/QALY BCR: 3–149 For different measures (+)	Most interventions are robust and remain cost-effective under various assumptions and conditions.
**Holland et al. (2014)** [78]	Health and climate benefits (VSL)	Performed but not specified	(1) Incremental costs shown in table in appendix for 2025B1: 222; B2: 1201; B6: 3339; B3: 4628; B4: 4679; MTRF: 47,006b for 2030; B7: 3334; MTRF: 50,681 (2) /	All EU member states achieve net benefits when switching from the CLE to the B3 scenario but not when switching to the MTFR scenario, except in the least-conservative mortality valuation scenario **(±)**	Lower VOLY has no effect on results.
**Srinivasan et al. (2018)** [79]	Health benefits (VSL)	/	(1) / (2) Avoided mortality/morbidity INR 962.222 crore.	INR 1.36 crore–INR 1.44 crore/life avoided **(+)**	/

/: not reported; BCR: benefit–cost ratio; GAINS: Greenhouse Gas–Air pollution Interactions and Synergies; ICER: incremental cost-effectiveness ratio; VOLY: value of a life year; VSL: value of statistical life; PSA: probabilistic sensitivity analysis; QALY: quality-adjusted life year; EU: European Union; CLE: current legislation; MTFR: maximum technically feasible reduction; INR: India Rupee. (+): only cost-effective results; (±): both cost-effective and non-cost-effective results, (blank): not mentioned.

## Supplementary Materials

The following supporting information can be downloaded at: https://www.mdpi.com/article/10.3390/ijerph22060926/s1. Table S1: Search string MEDLINE via PubMed; Table S2: Search string Web of Science Core Collection; Table S3: Search string EMBASE.

## Abbreviations

The following abbreviations are used in this manuscript:

CBA	Cost–benefit analysis
CBR	Cost–benefit ratio
CHEERS	Consolidated Health Economic Evaluation Reporting Standards
COI	Cost of illness
COPD	Chronic obstructive pulmonary disease
CUA	Cost–utility analysis
DALY	Disability-adjusted life year
IAM	Integrated assessment model
ICER	Incremental cost-effectiveness ratio
ICS	Improved cooking stoves
ICUR	Incremental cost–utility ratio
LPG	Liquid petroleum gas
MeSH	Medical Subject Heading
NB	Net benefits
QALY	Quality-adjusted life year
UK	United Kingdom
USA	United States of America
VSL	Value of statistical life
WHO	World Health Organization
WTP	Willingness to pay

## Appendix A

**Table A1:** Types of interventions found in the included studies.

**Residential**		
**Author (Publication Year)**	**Intervention**	**Comparator**
**Adibi et al. (2023)** [70]	Air purification: types of government reimbursement for air purifiers for residents with asthma.	Base case: the provincial government reimburses 100% of the cost of air purifiers for residents with asthma.
**Aunan et al. (2013)** [38]	Heating: replacement of biomass stoves with improved systems (implementation in individual households (1) or in the community (2) in households with (a) a chimney and without (b)).	Situation prior to the intervention.
**Cansino et al. (2019)** [62]	Implementation of solar panels and storage systems.	Business as usual: traditional wood-burning stoves.
**Feng et al. (2021)** [41]	Implementation of gas and electricity for heating.	Basic scenario without clean heating.
**Fisk et al. (2017)** [43]	Different methods to purify air with air purifiers (i1–i6).	Two basic scenarios: - Periodic air distribution system, low-efficiency filter, and no portable air purifier; - No air distribution system or portable air purifier.
**Fisk et al. (2017a)** [42]	Different methods to purify air with air purifiers (i1–i6).	Idem Fisk et al. (2017) [43].
**Irfan et al. (2021)** [45]	Implementation of stoves based on liquefied petroleum gases, gas, biogas, electricity, or improved cooking systems.	Not mentioned.
**Jeuland et al. (2016)** [32]	Improved wood-burning stoves, improved charcoal-burning stoves, implementation of stoves based on liquefied petroleum gases or electric stoves.	Business as usual: traditional wood-burning stoves.
**Liu Y. et al. (2021)** [47]	Different scenarios of air purification: S1: PM_2.5_ target = 35 µg/m^3^; S2: PM_2.5_ target = 25 µg/m^3^; S3: PM_2.5_ target = 15 µg/m^3^; S4: PM_2.5_ target = 10 µg/m^3^.	Base case: no air purifier.
**Mardones et al. (2021)** [33]	Implementation of kerosene heating with ventilation (1), pellet stove (2), certified wood-burning stove (4).	Most common method of heating prior to the intervention: wood.
**Meng et al. (2023)** [34]	Heating with electricity (1), gas (2), a combination of these two (3), coal in highly efficient stoves (4), pellet stove with gasifier (5).	Base case: Stated Policy Scenario.
**Meng et al. (2023b)** [49]	Heating with clean energy (not specified).	Base scenario without clean heating.
**Ramirez et al. (2024)** [69]	Two improved (biomass-improved cookstove tier 2 and tier 3) and three clean (LPG, biogas, and electricity induction).	Traditional cooking.
**Yang et al. (2024)** [36]	Air purifiers in different residential spaces: bedroom (1), kitchen (2), both (3), bedroom + living room (4), (4) + (2) (5), classroom (6).	Base scenario: no air purifier.
**Zhang et al. (2023)** [56]	Air purification with different target concentrations: S1–S4 → 5–15–25–35 µg/m^3^.	Base scenario, not specified.
**Industrial**		
**Author (publication year)**	**Intervention**	**Comparator**
**Buonocore et al. (2016)** [73]	Policy scenario similar to ‘Clean Power Plan’.	Business as usual, reference scenario.
**Chen et al. (2015)** [39]	Different environmental policy scenarios (FS: strict policy measure; FR: less strict policy measure).	Base case: development without implementation of environmental policy scenarios.
**Cropper et al. (2017)** [63]	Implementation of flue gas desulphurisation to reduce SO_2_.	No desulphurisation.
**Gao et al. (2016)** [44]	Policy scenario of coal savings, ‘end-of-pipe’ treatments, or an integrated scenario.	Base case: no coal savings, no emission reduction (counterfactual scenario).
**Guo et al. (2023)** [64]	Implementation of clean energy transition: early retirement of coal-fired units after 20 years of operation.	Base case: normal retirement of all energy facilities after 30 years.
**Levy et al. (2017)** [46]	Advanced-technology combined heat and power biomass system.	Counterfactual scenario: conventional configuration.
**Liu K et al. (2024)** [67]	(1) Ultralow APCDs (upgrading air pollutant control devices); (2) natural retirement of coal-fired industrial boilers (CFIBs); (3) early retirement of CFIBs; (4) enhanced retirement of CFIBs; (5) biomass replacement; (6) gas replacement.	Baseline scenario (2020).
**Thompson et al. (2016)** [52]	Two subnational carbon policy scenarios: ‘clean energy standard’, in which a fraction of electricity must be generated by clean energy, and ‘cap-and-trade method’ for emissions.	Base case: no carbon constraints.
**Wan et al. (2023)** [54]	Emission reduction strategies: dismantling small units, renovating existing units, promoting clean energy, building new units.	Not mentioned.
**Wiser et al. (2017)** [55]	Standards for renewable portfolio and its expansion.	‘Renewable Portfolio Standards’ purchase obligations were eliminated after 2014.
**Zhang et al. (2015)** [57]	Multipollutant strategy and gradual pollutant strategy.	No baseline scenario; both interventions are directly compared.
**Zhang et al. (2019)** [58]	Action Plan for Air Pollution Prevention and Control.	Not mentioned.
**Transport**		
**Author (publication year)**	**Intervention**	**Comparator**
**Ballinger et al. (2016)** [76]	Different measures to reduce transport emissions (e.g., low-emission zones, road closures, impact of noise barriers, etc.).	Scenario prior to the intervention.
**Evans et al. (2021)** [40]	Retrofitting heavy-duty commercial vehicles in use: diesel oxidation catalysts; diesel particulate filters.	Base case, not specified.
**Lomas et al. (2016)** [14]	Implementation of low-emission zones.	‘Do nothing’ scenario.
**Lopez-Aparicio et al. (2020)** [48]	Implementation of speed limits: Scenario 1, speed limit analysed with observed speeds; Scenario 2, speed limits analysed with maximum speeds.	Base case: no speed limit.
**Whitehurst et al. (2021)** [66]	Different scenarios for the expansion of cycling infrastructure: no increase, moderate increase, and significant increase in cyclists.	Base case, reference year.
**Zhou et al. (2019)** [60]	Replacement of labelled vehicles.	Base case: no policy.
**Zhou et al. (2022)** [61]	Limitations for high-emission vehicles.	Base case: the amount of current high-emission-vehicle-restricted areas without the policy.
**Agricultural**		
**Author (publication year)**	**Intervention**	**Comparator**
**Giannadaki et al. (2018)** [74]	(1) Implementation of low-nitrogen feed; (2) low-emission animal housing; (3) fertiliser storage capacity; (4) techniques to reduce fertiliser emissions.	Not mentioned.
**Giannakis et al. (2019)** [30]	(1) Implementation of low-nitrogen feed; (2) manure storage capacity; (3) low-emission animal housing; (4) replacement or improvement of urea fertiliser.	Control simulation, not further specified.
**Liu M. et al. (2019)** [75]	Reduction in NH_3_ and SO_2_ emissions (not specified).	Emissieniveaus in 2015.
**Wagner et al. (2015)** [35]	(1) Calcium ammonium nitrate instead of urea fertiliser; (2) reduced tillage; (3) low-protein feed for pigs and poultry; (4) covering techniques for manure storage; (5) techniques for manure spreading; (6) air purification systems for exhaust gases.	Base case: estimated emissions under current conditions.
**Wagner et al. (2017)** [53]	Scenario where emission reduction strategies are implemented on every farm.	Reference scenario: estimated emissions under current reduction measures.
**Zhang et al. (2020)** [37]	Change in diet, optimal nitrogen use, and less agricultural waste through better recycling of animal manure, crop residues, and human waste.	Base case: current policy measures and plans.
**Intersectoral**		
**Author (publication year)**	**Intervention**	**Comparator**
**Lavee et al. (2018)** [65]	Twenty different policy measures in the energy, industry, transport, and household sectors.	Baseline scenario: current policy measures led by IMoEP and other relevant government departments.
**Miranda et al. (2016)** [50]	Reduction in emissions through a combination of the following interventions: (1) HYB, replacement of 10% of vehicles under EURO3 with hybrid cars; (2) LEZ, low emission zone in Porto; (3) FIR, replacement/conversion of 50% of conventional open fireplaces with more efficient equipment; (4) IND, application of environmentally friendly technology that causes a 10% reduction in PM_10_ emissions in production processes and industrial combustion.	Reference scenario where the emissions reflect the already implemented measures.
**Schucht et al. (2018)** [51]	Different measures in the energy, transport, industrial, and household sectors.	Not mentioned.
**Zhao et al. (2022)** [59]	Introduction of local and national action plans to reduce air pollution.	Current air pollution level.
**No real intervention**		
**Author (publication year)**	**Intervention**	**Comparator**
**Amann et al. (2017)** [77]	Different emission standards	Not reported.
**Holland et al. (2014)** [78]	Maximum Technically Feasible Reduction (MTFR) scenario and a series of intermediate scenarios for 2025 and 2030: These scenarios vary in the ambition levels set for mortality linked to PM_2.5_ exposure, ozone, and eutrophication.	Scenario with current legislation.
**Howard et al. (2019)** [31]	Three different scenarios: Scenario 1: The PM_10_ emission standard is reduced from 28.15 g/kWh to 0.69 g/kWh; Scenario 2: The PM_10_ emission standard is reduced from 0.69 g/kWh to 0.36 g/kWh; Scenario 3: The PM_10_ emission standard is reduced from 0.36 g/kWh to 0.04 g/kWh.	Base case, not specified.
**Kim et al. (2020)** [71]	Two scenarios (no real intervention): Keep the standard as is; Adopt and enforce US emissions standards (12 µg/m^3^).	- PM_2.5_ emissions in Italy (2015): 13 µg/m^3^; - PM_2.5_ emission in France (2015): 19 µg/m^3^.
**Liu Z et al. (2023)** [68]	Nox-only, Nox-AVOC-A, Nox-AVOC-B	Not specified.
**Schmitt et al. (2016)** [72]	A hypothetical scenario of an immediate reduction of 1 µg/m^3^ in the average ambient concentration of PM_2.5_.	No intervention.
**Srinivasan et al. (2018)** [79]	Different emission standards	Not reported.

## Appendix B

**Table A2:** Quality assessment of the included studies. Part 1.

	Adibi et al. (2023) [70]	Aunan et al. (2013) [77]	Buonocore et al. (2016) [73]	Cansino et al. (2019) [62]	Chen et al. (2015) [39]	Cropper et al. (2017) [63]	Evans et al. (2021) [40]	Feng et al. (2021) [41]	Fisk et al. (2017) [43]	Fisk et al. (2017a) [42]	Gao et al. (2016) [44]	Giannadaki et al. (2018) [74]	Giannakis et al. (2019) [30]	Guo et al. (2023) [64]	Howard et al. (2019) [31]	Irfan et al. (2021) [45]	Jeuland et al. (2016) [32]	Kim et al. (2021) [71]	Lavee et al. (2018) [65]	Levy et al. (2017) [46]	Liu K et al. (2024) [67]	Liu M. et al. (2019) [75]
**(1) Title**	✔	✔	±	x	±	✔	±	±	✔	✔	±	x	✔	±	✔	✔	✔	✔	✔	✔	±	±
**(2) Abstract**	✔	✔	✔	✔	✔	✔	✔	✔	✔	✔	✔	✔	✔	✔	✔	✔	✔	✔	±	✔	✔	±
**(3) Background and objectives**	✔	✔	✔	✔	✔	✔	✔	✔	✔	✔	✔	✔	✔	✔	✔	✔	✔	✔	✔	✔	✔	✔
**(4) Health economic analysis plan**	✔	x	x	x	x	x	x	x	x	x	x	x	x	x	x	x	x	x	x	x	x	x
**(5) Study population**	✔	✔	x	x	x	x	✔	x	x	x	x	x	✔	x	x	x	x	x	x	x	x	x
**(6) Setting and location**	✔	✔	✔	✔	✔	✔	✔	✔	✔	✔	✔	✔	✔	✔	✔	✔	✔	✔	✔	✔	✔	✔
**(7) Comparators**	✔	✔	✔	✔	✔	✔	✔	✔	✔	✔	✔	✔	✔	✔	✔	✔	✔	✔	✔	✔	✔	✔
**(8) Perspective**	✔	x	x	x	x	x	x	✔	✔	x	✔	✔	x	x	x	x	✔	✔	x	x	x	x
**(9) Time horizon**	✔	x	x	x	x	✔	x	✔	x	x	✔	x	x	x	x	✔	✔	✔	x	✔	✔	x
**(10) Discount rate**	✔	✔	✔	x	✔	✔	✔	x	x	x	✔	x	x	x	x	✔	✔	✔	x	✔	x	x
**(11) Selection of outcomes**	✔	✔	✔	✔	✔	✔	✔	✔	✔	✔	✔	✔	✔	✔	✔	✔	✔	✔	✔	✔	✔	✔
**(12) Measurement of outcomes**	✔	✔	✔	✔	x	✔	✔	✔	✔	✔	✔	✔	✔	✔	✔	✔	✔	✔	✔	✔	✔	✔
**(13) Valuation of outcomes**	✔	✔	✔	✔	✔	✔	✔	✔	✔	✔	✔	✔	✔	✔	✔	✔	✔	✔	✔	x	x	✔
**(14) Measurement and valuation of resources and costs**	✔	✔	✔	✔	✔	✔	✔	✔	✔	✔	✔	x	✔	✔	✔	✔	✔	✔	✔	✔	✔	✔
**(15) Currency, price date, and conversion**	✔	✔	✔	✔	✔	✔	x	x	x	x	✔	x	x	✔	✔	✔	x	✔	x	x	✔	x
**(16) Rationale and description of model**	✔	x	✔	x	✔	✔	x	NA	NA	✔	✔	✔	✔	✔	✔	x	x	✔	✔	✔	✔	✔
**(17) Analytics and assumptions**	✔	x	✔	x	✔	x	✔	NA	NA	x	✔	✔	✔	✔	✔	x	✔	✔	x	✔	✔	✔
**(18) Characterising heterogeneity**	x	x	x	x	x	x	x	x	x	x	x	x	x	x	x	x	x	x	x	x	x	x
**(19) Characterising distributional effects**	x	x	x	x	x	x	x	x	x	x	x	x	x	x	x	x	x	x	x	x	x	x
**(20) Characterising uncertainty**	✔	✔	x	x	x	✔	✔	x	x	✔	✔	✔	x	✔	✔	✔	✔	✔	x	x	x	x
**(21) Approach to engagement with patients and others affected by the study**	x	x	x	x	x	x	x	x	x	x	x	x	x	x	x	x	x	x	x	x	x	x
**(22) Study parameters**	✔	✔	✔	✔	✔	✔	✔	✔	✔	✔	✔	✔	✔	✔	✔	✔	✔	✔	✔	✔	✔	✔
**(23) Summary of the main results**	✔	✔	✔	✔	✔	✔	✔	✔	✔	✔	✔	✔	✔	✔	✔	✔	✔	✔	✔	✔	✔	✔
**(24) Effect of uncertainty**	✔	✔	x	x	x	✔	✔	x	x	✔	✔	✔	x	✔	✔	✔	✔	✔	x	x	x	x
**(25) Effect of engagement with patients and others affected by the study**	x	x	x	x	x	x	x	x	x	x	x	x	x	x	x	x	x	x	x	x	x	x
**(26) Study findings, limitations, generalizability, and current knowledge**	✔	✔	✔	✔	✔	✔	✔	✔	✔	✔	✔	✔	✔	✔	✔	✔	✔	✔	x	✔	✔	✔
**(27) Source of funding**	x	✔	x	✔	x	x	x	✔	✔	✔	✔	x	✔	x	x	x	x	✔	x	✔	✔	✔
**(28) Conflict of interest**	x	x	x	✔	x	x	x	✔	✔	✔	x	✔	✔	✔	x	x	x	✔	✔	x	✔	x
**TOTAL/28**	22	18	15.5	14	14.5	18	16.5	15.5	15	17	20.5	16	17	17.5	17	17	18	22	12.5	16	16.5	14

‘x’ is counted as ‘0’; ‘✔’ is counted as ‘1’; ‘±’ is counted as ‘0.5’; NA: not applicable.

**Table A3:** Quality assessment of the included studies. Part 2.

	Liu Y. et al. (2021) [47]	Liu Z et al. (2023) [68]	Lomas et al. (2016) [14]	Lopez-Aparicio et al. (2020) [48]	Mardones et al. (2021) [33]	Meng et al. (2023) [34]	Meng et al. (2023) [49]	Miranda et al. (2016) [50]	Ramirez et al. (2024) [69]	Schmitt L.H.M. (2016) [72]	Schucht et al. (2018) [51]	Thompson et al. (2016) [52]	Wagner et al. (2015) [35]	Wagner et al. (2017) [53]	Wan et al. (2023) [54]	Whitehurst et al. (2021) [66]	Wiser et al. (2017) [55]	Yang et al. (2024) [36]	Zhang et al. (2015) [57]	Zhang et al. (2019) [58]	Zhang et al. (2021) [37]	Zhang et al. (2023) [56]	Zhao et al. (2022) [59]	Zhou et al. (2019) [60]	Zhou et al. (2022) [61]
**(1) Title**	✔	✔	✔	✔	✔	✔	✔	±	✔	✔	✔	±	✔	✔	✔	✔	✔	✔	±	✔	✔	✔	x	✔	✔
**(2) Abstract**	✔	±	✔	✔	✔	±	✔	✔	✔	✔	✔	✔	✔	✔	✔	✔	✔	✔	±	✔	±	✔	✔	±	✔
**(3) Background and objectives**	✔	✔	✔	✔	✔	✔	✔	✔	✔	✔	✔	✔	✔	✔	✔	✔	✔	✔	✔	✔	✔	✔	✔	✔	✔
**(4) Health economic analysis plan**	x	x	x	x	x	x	x	x	x	x	x	x	x	x	x	x	x	x	x	x	x	x	x	x	x
**(5) Study population**	x	x	✔	x	✔	✔	x	x	x	✔	x	x	x	x	x	x	x	x	x	x	x	✔	x	x	x
**(6) Setting and location**	✔	✔	✔	✔	✔	✔	✔	✔	✔	✔	✔	✔	✔	✔	✔	✔	✔	✔	✔	✔	✔	✔	✔	✔	✔
**(7) Comparators**	✔	✔	✔	✔	✔	✔	✔	✔	✔	✔	✔	✔	✔	✔	✔	✔	✔	✔	✔	✔	✔	✔	✔	✔	✔
**(8) Perspective**	x	✔	✔	x	x	x	x	x	✔	x	x	x	✔	✔	x	✔	x	x	x	✔	x	x	x	✔	✔
**(9) Time horizon**	x	✔	✔	x	x	x	x	x	✔	✔	x	x	x	✔	x	✔	✔	x	✔	✔	✔	x	✔	✔	✔
**(10) Discount rate**	x	x	✔	x	✔	✔	✔	x	✔	✔	x	x	x	✔	x	✔	✔	x	✔	x	✔	x	x	✔	✔
**(11) Selection of outcomes**	✔	✔	✔	✔	✔	✔	✔	✔	✔	✔	✔	✔	x	✔	✔	✔	✔	✔	✔	✔	✔	✔	✔	✔	✔
**(12) Measurement of outcomes**	✔	✔	✔	✔	✔	✔	✔	✔	✔	✔	✔	✔	✔	✔	✔	✔	✔	✔	✔	✔	✔	✔	✔	✔	✔
**(13) Valuation of outcomes**	✔	✔	✔	✔	✔	✔	✔	✔	✔	✔	✔	✔	x	✔	✔	x	x	✔	x	✔	✔	✔	✔	✔	✔
**(14) Measurement and valuation of resources and costs**	✔	✔	x	✔	✔	✔	✔	✔	✔	✔	x	✔	±	✔	✔	✔	✔	✔	✔	✔	✔	✔	x	✔	✔
**(15) Currency, price date, and conversion**	x	✔	x	✔	x	✔	✔	✔	✔	✔	x	✔	x	✔	✔	✔	x	x	x	✔	✔	x	x	✔	✔
**(16) Rationale and description of model**	✔	✔	x	✔	NA	✔	✔	✔	✔	✔	✔	✔	✔	✔	✔	✔	✔	✔	✔	x	✔	✔	NA	✔	✔
**(17) Analytics and assumptions**	✔	✔	x	x	NA	✔	x	✔	✔	✔	x	x	✔	x	✔	✔	✔	✔	x	x	✔	x	NA	✔	✔
**(18) Characterising heterogeneity**	x	x	x	x	x	x	x	x	x	x	x	x	x	x	x	x	x	x	x	x	x	x	x	x	x
**(19) Characterising distributional effects**	x	x	x	x	x	x	x	x	x	x	x	x	x	x	x	x	x	x	x	x	x	x	x	x	x
**(20) Characterising uncertainty**	✔	x	x	✔	✔	✔	✔	x	x	✔	x	✔	x	✔	x	✔	✔	✔	✔	x	x	✔	x	x	x
**(21) Approach to engagement with patients and others affected by the study**	x	x	x	x	x	x	x	x	x	x	x	x	x	x	x	x	x	x	x	x	x	x	x	x	x
**(22) Study parameters**	✔	✔	✔	✔	✔	✔	✔	✔	✔	✔	✔	✔	✔	✔	✔	✔	✔	✔	✔	✔	✔	✔	✔	✔	✔
**(23) Summary of the main results**	✔	x	✔	✔	✔	✔	✔	✔	✔	✔	✔	✔	✔	✔	✔	✔	✔	✔	✔	✔	✔	✔	✔	✔	✔
**(24) Effect of uncertainty**	✔	x	x	✔	✔	✔	✔	x	x	✔	x	x	✔	✔	x	✔	x	x	✔	x	x	✔	x	x	x
**(25) Effect of engagement with patients and others affected by the study**	**x**	x	x	x	x	x	x	x	x	x	x	x	x	x	x	x	x	x	x	x	x	x	x	x	x
**(26) Study findings, limitations, generalizability, and current knowledge**	✔	✔	✔	✔	✔	✔	✔	✔	✔	✔	✔	✔	✔	✔	✔	✔	✔	✔	✔	✔	±	✔	✔	✔	✔
**(27) Source of funding**	✔	✔	✔	✔	x	x	✔	✔	x	x	✔	✔	x	✔	✔	✔	✔	x	✔	x	x	✔	✔	✔	✔
**(28) Conflict of interest**	✔	✔	✔	✔	✔	✔	✔	x	✔	x	✔	x	x	x	✔	✔	x	✔	x	x	✔	✔	✔	x	✔
**TOTAL/28**	**18**	**18.5**	**17**	**18**	**17**	**19.5**	**19**	**15.5**	**19**	**20**	**14**	**15.5**	**13.5**	**20**	**17**	**21**	**17**	**16**	**16**	**15**	**17**	**18**	**13**	**18.5**	**20**

‘x’ is counted as ‘0’; ‘✔’ is counted as ‘1’; ‘±’ is counted as ‘0.5’; NA: not applicable.

**Table A4:** Quality assessment of the included grey literature.

	Amann et al. (2017) [77]	Ballinger et al. (2016) [76]	Holland et al. (2014) [78]	Srinivasan et al. (2018) [79]
**(1) Title**	✔	±	✔	✔
**(2) Abstract**	±	✔	✔	✔
**(3) Background and objectives**	✔	✔	✔	✔
**(4) Health economic analysis plan**	x	x	x	x
**(5) Study population**	x	x	x	x
**(6) Setting and location**	✔	✔	✔	✔
**(7) Comparators**	x	✔	✔	x
**(8) Perspective**	✔	✔	x	x
**(9) Time horizon**	✔	✔	✔	✔
**(10) Discount rate**	x	✔	x	✔
**(11) Selection of outcomes**	✔	✔	✔	✔
**(12) Measurement of outcomes**	✔	✔	✔	✔
**(13) Valuation of outcomes**	✔	✔	✔	✔
**(14) Measurement and valuation of resources and costs**	✔	✔	✔	✔
**(15) Currency, price date, and conversion**	✔	±	✔	✔
**(16) Rationale and description of model**	x	✔	✔	✔
**(17) Analytics and assumptions**	x	✔	✔	✔
**(18) Characterising heterogeneity**	x	x	x	x
**(19) Characterising distributional effects**	x	x	x	x
**(20) Characterising uncertainty**	✔	✔	x	x
**(21) Approach to engagement with patients and others affected by the study**	x	x	x	x
**(22) Study parameters**	✔	✔	✔	✔
**(23) Summary of the main results**	✔	✔	✔	✔
**(24) Effect of uncertainty**	✔	x	✔	x
**(25) Effect of engagement with patients and others affected by the study**	x	x	x	x
**(26) Study findings, limitations, generalizability, and current knowledge**	✔	✔	✔	✔
**(27) Source of funding**	x	✔	✔	✔
**(28) Conflict of interest**	x	x	x	x
**TOTAL/28**	15.5	19	18	17

‘x’ is counted as ‘0’; ‘✔’ is counted as ‘1’; and ‘±’ is counted as ‘0.5’.
