# Supplementary material for: Evaluating the Cost-Effectiveness of Air Pollution Mitigation Strategies: A Systematic Review
Source: Int J Environ Res Public Health. 2025 Jun 11;22(6):926. doi: 10.3390/ijerph22060926 (PMC12192989; doi:10.3390/ijerph22060926)
Supplement: Supplementary file 1 [file ijerph-22-00926-s001.zip › ijerph-3528358-supplementary.pdf]

# Supplementary material: Search strategy literature review

## Database 1: MEDLINE via Pubmed

**Table S1**: Search string MEDLINE via PubMed

| Keywords (MeSH) | Query |
|---|---|
| **Air pollution, air quality, particulate matter** | "Air Pollution"[Mesh] OR "Air Quality"[tiab] OR “air pollut*”[tiab] OR “atmospheric pollut*”[tiab] OR “air contaminant*”[tiab] OR “polluted air”[tiab] OR “air quality”[tiab] OR “outdoor air pollut*”[tiab] OR “ambient air pollut*”[tiab] OR “atmospheric pollut*”[tiab] OR “Particulate Matter”[Mesh] OR "Particulate matter"[tiab] OR “airborne particulate matter”[tiab] OR “particulate air pollutants”[tiab] OR “ambient particulate matter”[tiab] OR “ultrafine particulate matter”[tiab] OR “ultrafine particles”[tiab] |
| **Cost-effectiveness, cost-benefit analyse, cost-utility, health economic, cost-analyse, QALY, quality-adjusted life year, DALY, disability-adjusted life year, health impact/outcome/gain** | "Cost-Benefit Analysis"[Mesh] OR "Cost-Benefit Analysis"[tiab] OR “cost utilit*”[tiab] OR “costutilit*”[tiab] OR "Cost Benefit"[tiab] OR "Costs and Benefits"[tiab] OR "Benefits and Costs"[tiab] OR "Benefit and Cost"[tiab] OR "Cost and Benefit"[tiab] OR "Marginal Analys*"[tiab] OR "Cost Benefit Data"[tiab] OR “economic evaluation*”[tiab]<br>OR<br>"Cost-Effectiveness Analysis"[Mesh] OR "Cost Effectiveness"[tiab] OR “ICER”[tiab] OR “incremental cost-effectiveness ratio”[tiab] OR “incremental cost utility ratio”[tiab] OR “ICUR”[tiab]<br>OR<br>“cost minimization”[tiab] OR “costminimization”[tiab] OR “cost efficien*”[tiab] OR “costefficien*”[tiab] OR “health economic”[tiab] OR “healtheconomic”[tiab] |
| **Intervention, strategy, assessment, policy, management, abatement, mitigation, reduction** | “Intervention*”[tiab] OR “strateg*”[tiab] OR “reduction”[tiab] OR “mitigation*”[tiab] OR “assessment*”[tiab] OR “policy”[tiab] OR “policies”[tiab] OR “abatement*”[tiab] OR “management”[tiab] |

## Database 2: Web of Science Core Collection via Web of Science

**Table S2**: Search string Web of Science Core Collection

| Keywords | Query |
|---|---|
| **Air pollution, air quality, particulate matter** | TS=("Air Pollution" OR "Air Quality" OR "air pollut*" OR “atmospheric pollut*" OR "air contaminant*" OR "polluted air" OR "air quality" OR "outdoor air pollut*" OR "ambient air pollut*" OR "atmospheric pollut*" OR "Particulate Matter" OR "Particulate matter" OR “airborne particulate matter” OR “particulate air pollutants” OR “ambient particulate matter” OR “ultrafine particulate matter” OR “ultrafine particles”) |
| **Cost-effectiveness, cost-benefit analyse, cost-utility, health economic, cost-analyse, QALY, quality-adjusted life year, DALY, disability-adjusted life year** | TS=("Cost-Benefit Analysis" OR "Cost-Benefit Analysis" OR "cost utilit*" OR “costutilit*” OR "Cost Benefit" OR "Costs and Benefits" OR "Benefits and Costs" OR "Benefit and Cost" OR "Cost and Benefit" OR "Marginal Analys*" OR "Cost Benefit Data" OR "economic evaluation*"<br>OR<br>"Cost-Effectiveness Analysis" OR "Cost Effectiveness" OR “ICER” OR "incremental cost-effectiveness ratio" OR "incremental cost utility ratio" OR “ICUR”<br>OR |

| | |
|---|---|
| | "cost minimization" OR “costminimization” OR "cost efficien*" OR “costefficien*” OR "health economic" OR “healtheconomic” |
| **Intervention, strategy, assessment, policy, management, abatement, mitigation, reduction** | TS=(“Intervention*” OR “strateg*” OR “reduction” OR “mitigation*” OR “assessment*” OR “policy“ OR “policies” OR “abatement*” OR “management”) |

## Database 3: EMBASE

**Table S3**: Search string EMBASE

| Keywords (Emtree) | Query |
|---|---|
| **Air pollution, air quality, particulate matter** | 'Air Pollution'/exp OR 'Air Quality':ti,ab,kw OR 'air pollut*':ti,ab,kw OR 'atmospheric pollut*':ti,ab,kw OR 'air contaminant*':ti,ab,kw OR 'polluted air':ti,ab,kw OR 'air quality':ti,ab,kw OR 'outdoor air pollut*':ti,ab,kw OR 'ambient air pollut*':ti,ab,kw OR 'atmospheric pollut*':ti,ab,kw OR 'Particulate Matter'/exp OR 'Particulate matter':ti,ab,kw OR 'airborne particulate matter':ti,ab,kw OR 'particulate air pollutants':ti,ab,kw OR 'ambient particulate matter':ti,ab,kw OR 'ultrafine particulate matter':ti,ab,kw OR 'ultrafine particles':ti,ab,kw |
| **Cost-effectiveness, cost-benefit analyse, cost-utility, health economic, cost-analyse, QALY, quality-adjusted life year, DALY, disability-adjusted life year** | 'Cost-Benefit Analysis'/exp OR 'Cost-Benefit Analysis':ti,ab,kw OR 'cost utilit*':ti,ab,kw OR ‘costutilit*’:ti,ab,kw OR 'Cost Benefit':ti,ab,kw OR 'Costs and Benefits':ti,ab,kw OR 'Benefits and Costs':ti,ab,kw OR 'Benefit and Cost':ti,ab,kw OR 'Cost and Benefit':ti,ab,kw OR 'Marginal Analys*':ti,ab,kw OR 'economic evaluation*':ti,ab,kw<br>OR<br>'Cost-Effectiveness Analysis'/exp OR 'Cost Effectiveness':ti,ab,kw OR ICER:ti,ab,kw OR 'incremental cost-effectiveness ratio':ti,ab,kw OR ‘incremental cost utility ratio’:ti,ab,kw OR ‘ICUR’:ti,ab,kw<br>OR<br>'cost minimization':ti,ab,kw OR ‘costminimization’:ti,ab,kw OR 'cost efficien*':ti,ab,kw OR ‘costefficien*’:ti,ab,kw OR 'health economic':ti,ab,kw OR ‘healtheconomic’:ti,ab,kw |
| **Intervention, strategy, assessment, policy, management, abatement, mitigation, reduction** | ‘Intervention*’:ti,ab,kw OR ‘strateg*’:ti,ab,kw OR ‘reduction’:ti,ab,kw OR ‘mitigation*’:ti,ab,kw OR ‘assessment*’:ti,ab,kw OR ‘policy’:ti,ab,kw OR ‘policies’:ti,ab,kw OR ‘abatement*’:ti,ab,kw OR ‘management’:ti,ab,kw |
